# Natural products for anti-fibrotic therapy in idiopathic pulmonary fibrosis: marine and terrestrial insights

**DOI:** 10.3389/fphar.2025.1524654

**Published:** 2025-05-14

**Authors:** Meiting Ma, Zhengqi Chu, Hongyu Quan, Hanxu Li, Yuran Zhou, Yanhong Han, Kefeng Li, Wenjun Pan, De-Yun Wang, Yan Yan, Zunpeng Shu, Yongkang Qiao

**Affiliations:** ^1^ Key Laboratory of Cell Proliferation and Regulation Biology, Ministry of Education, Faculty of Arts and Sciences, Beijing Normal University, Zhuhai, China; ^2^ Guangdong-Hong Kong-Macao University Joint Laboratory of Interventional Medicine, The Fifth Affiliated Hospital, Sun Yat-Sen University, Zhuhai, China; ^3^ Faculty of Applied Sciences, Macao Polytechnic University, Macau, Macao SAR, China; ^4^ Department of Oncology, The Third Affiliated Hospital of Shenzhen University, Shenzhen, China; ^5^ Department of Otolaryngology, Yong Loo Lin School of Medicine, National University Health System, National University of Singapore, Singapore, Singapore

**Keywords:** anti-fibrotic, natural products, idiopathic pulmonary fibrosis, therapeutic target, mechanisms of action

## Abstract

Idiopathic Pulmonary Fibrosis (IPF) is a chronic fibrotic interstitial lung disease (ILD) of unknown etiology, characterized by increasing incidence and intricate pathogenesis. Current FDA-approved drugs suffer from significant side effects and limited efficacy, highlighting the urgent need for innovative therapeutic agents for IPF. Natural products (NPs), with their multi-target and multifaceted properties, present promising candidates for new drug development. This review delineates the anti-fibrotic pathways and targets of various natural products based on the established pathological mechanisms of IPF. It encompasses over 20 compounds, including flavonoids, saponins, polyphenols, terpenoids, natural polysaccharides, cyclic peptides, deep-sea fungal alkaloids, and algal proteins, sourced from both terrestrial and marine environments. The review explores their potential roles in mitigating pulmonary fibrosis, such as inhibiting inflammatory responses, protecting against lipid peroxidation damage, suppressing mesenchymal cell activation and proliferation, inhibiting fibroblast migration, influencing the synthesis and secretion of pro-fibrotic factors, and regulating extracellular matrix (ECM) synthesis and degradation. Additionally, it covers various *in vivo* and *in vitro* disease models, methodologies for analyzing marker expression and signaling pathways, and identifies potential new therapeutic targets informed by the latest research on IPF pathogenesis, as well as challenges in bioavailability and clinical translation. This review aims to provide essential theoretical and technical insights for the advancement of novel anti-pulmonary fibrosis drugs.

## 1 Introduction

Pulmonary fibrosis (PF) is a progressive lung disease characterized by high mortality, abnormal proliferation of lung fibroblasts, inflammatory damage, tissue destruction, and scarring (Thannickal et al., 2004). Based on etiology, PF can be classified into idiopathic pulmonary fibrosis (IPF) and progressive pulmonary fibrosis (PPF). IPF, the most common idiopathic form, is defined by a radiological or histopathological pattern of usual interstitial pneumonia (UIP) without an identifiable cause. In contrast, PPF refers to progressive fibrotic changes observed in non-IPF interstitial lung diseases (ILDs)—such as connective tissue disease-associated ILD or hypersensitivity pneumonitis—evidenced by declining lung function, worsening symptoms, or radiological progression. Although both subtypes lead to irreversible lung damage, they differ distinctly in clinical context and management strategies. Key distinctions include etiology (IPF is idiopathic, whereas PPF is secondary to an underlying ILD), diagnostic criteria (IPF is identified by characteristic UIP patterns, while PPF is defined by documented disease progression), and therapeutic approaches (IPF is primarily managed with antifibrotic agents such as nintedanib and pirfenidone, whereas PPF typically requires a combination of disease-specific interventions and antifibrotic therapy) (Koudstaal et al., 2023; Liu et al., 2022a). In this review, we will focus specifically on the pathogenesis and emerging therapeutic strategies targeting IPF, given the fact that IPF represents the majority of PF cases and poses unique treatment challenges.

IPF predominantly affects individuals over 60 years and is more common in men. Early-stage IPF is typically asymptomatic and lacks simple, user-friendly screening tools for early detection, often leading to diagnosis at an advanced stage. Moreover, the absence of clinically applicable biomarkers to guide treatment throughout the disease course, coupled with limited therapeutic options and a median survival of only 3–5 years post-diagnosis, underscores the urgent need for better diagnostic and therapeutic strategies (Koudstaal et al., 2023; Bonella et al., 2023). The pathogenesis of IPF involves complex molecular interactions that contribute to its poor prognosis and limited survival. As summarised in Table 1, although first-generation antifibrotic drugs, such as pirfenidone and nintedanib—approved over a decade ago—have demonstrated efficacy in slowing IPF progression and improving survival rates in clinical trials, their benefits are constrained by significant side effects (e.g., nausea, diarrhea, and photosensitivity) that compromise long-term tolerability (King et al., 2011; Maher, 2024). Moreover, although multiple anti-IPF drugs in the preclinical research stage (Table 2) reduce the rate of lung function decline in clinical studies (Maher, 2024; Lamb, 2021; King et al., 2014), they fail to halt or reverse fibrosis progression, thereby highlighting the urgent demand for innovative therapeutic approaches (Flaherty et al., 2019; Behr et al., 2021).

**TABLE 1:** Summary of FDA-approved drugs for idiopathic pulmonary fibrosis.

Clinical drug name	Chemical structure	Source (synthesis/extraction)	Efficacy	Untoward effect	Drug/mechanism of action
Pirfenidone	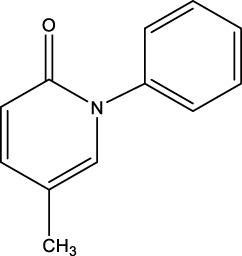	Synthesis: an indolinone derivative that was derived from a chemical lead optimization program designed for receptor tyrosine kinase inhibitors (Patent application WO2001027081, example 473)	Associated with a reduction in the relative risk of mortality compared with placebo over 120 weeks (Nathan et al., 2017)	Frequently associated with gastrointestinal symptoms (primarily nausea) and skin-related events (rash and photosensitivity) (King et al., 2014)	Downregulation of key pro-fibrotic growth factors, including TGF-β, and inhibited the production of inflammatory cytokines such as tumor necrosis factor-α (Nathan et al., 2017)
Nintedanib	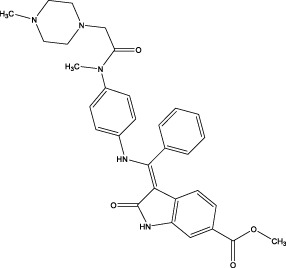	Synthesis: a small molecule tyrosine kinase inhibitor, developed by the German pharmaceutical company Boehringer Ingelheim	In patients with progressive fibrosing interstitial lung diseases, the annual decline in FVC was significantly lower in those receiving nintedanib compared to those on placebo (Flaherty et al., 2019)	The main adverse effects of nintedanib include gastrointestinal issues and liver function abnormalities, with diarrhea affecting about two-thirds of patients, followed by nausea, vomiting, and weight loss (Rugo et al., 2019) (Flaherty et al., 2019)	An intracellular inhibitor that targets multiple tyrosine kinases, including VEGF, FGF, and PDGF receptors (Hilberg et al., 2008) (Liu et al., 2022a; Spagnolo et al., 2021)

**TABLE 2:** Ongoing randomized clinical trials of antifibrotic drugs for treatment of idiopathic pulmonary fibrosis.

Clinical drug name	Chemical structure	Source (synthesis/extraction)	Efficacy	Phase	Untoward effect	Drug/mechanism of action
Saracatinib	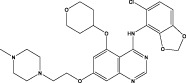	Synthesis	In Preclinical Models (include vitro human lung fibroblasts, mouse models, and human *ex vivo* lung slice models, the effectiveness of saracatinib in blocking fibrogenic responses was equal or superior to nintedanib and pirfenidone (Ahangari et al., 2022)	Phase 1b/2a clinical trial (from US Clinical Trials Registry)	Not yet reported	a potent and selective Src kinase inhibitor, originally developed for oncological indications (Baselga et al., 2010; Chang et al., 2008),also can effect epithelial–mesenchymal transition, TGF-β, and WNT signaling (Ahangari et al., 2022; Rugo et al., 2019)
PBI-4050	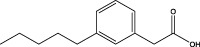	Synthesis	Reduction in FVC decline in the treatment group compared with placebo. Hepatobiliary SAE (early termination of study) (Khalil et al., 2019)	Phase 2 (NCT02538536)	Diarrhea was the most common adverse event, but its incidence rate was significantly lower than that of the approved drugs of the same type	Acts as an orally active agonist for GPR40 and as an antagonist or inverse agonist for GPR84
BMS-986278	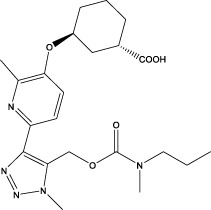	Synthesis	In *post hoc* analyses, treatment with BMS-986020 led to significant improvements in QLF (defined as a ≥2% reduction in QLF score) on HRCT and significant reductions in biomarkers of extracellular matrix turnover from baseline to week 26 relative to placebo (Corte et al., 2021)	Phase 2 (NCT04308681)	Hepatosis	A lysophosphatidic acid receptor 1 (LPA) antagonist
Nerandomilast (BI 1015550)	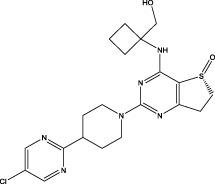	Synthesis	Treatment with BI 1015550, either alone or with background use of an antifibrotic agent, prevented a decrease in lung function in patients with idiopathic pulmonary fibrosis (Richeldi et al., 2022)	Phase 2 (NCT04419506)	Diarrhea	An oral preferential inhibitor of the PDE4B subtype
Bexotegrast (PLN 74809)	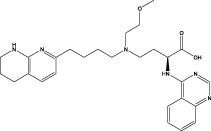	Synthesis	A dose-dependent antifibrotic effect of bexotegrast was observed with QLF imaging, and a decrease in fibrosis-associated biomarkers was observed with bexotegrast versus placebo (Lancaster et al., 2024)	Phase 2a (NCT04396756)	Hepatosis	A small molecule dual selective inhibitor with activity targetingαVβ1andαVβ6
AP01 (aerosolized pirfenidone)	Same as pirfenidone	Synthesis	Mean FVC % predicted remained stable in the 100 mg two times per day group (West et al., 2023)	Phase 1b ACTRN12618001838202	Adverse effects less frequent with AP01 than with oral pirfenidone in other clinical trials	Same as pirfenidone
TD139	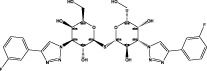	Synthesis	Inhaled TD139 safe and well tolerated in healthy subjects and IPF patients (Hirani et al., 2021)	Phase 1/2a (NCT02257177)	The most commonly occurring TEAE associated with TD139 was mild dysgeusia (distortion of sense of taste) (36.1%)	Can suppress Gal-3 expression on bronchoalveolar lavage macrophages and, in a concerted fashion, decrease plasma biomarkers associated with IPF progression
PA101	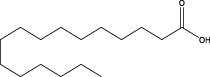	Synthesis	Day-time cough reduction in IPF at day 14 in the treatment group compared with the placebo group (Birring et al., 2017)	Phase 2 (NCT02412020)	No SAE observed	A novel high-concentration formulation of sodium cromoglicate

Compared to traditional synthetic drugs, natural products (NPs) exhibit higher structural diversity and complexity, characterized by a greater number of SP3-hybridized carbon atoms (Atanasov et al., 2021). Advances in the study of NPs have highlighted their favorable pharmacological profiles and broad biological activities, positioning them as significant lead compounds for new drug development. Research efforts are increasingly focused on the biological activity screening, drug targets, mechanisms of action, and drugability of NPs, marking a pivotal direction in pharmaceutical research (Haque et al., 2022). Cutting-edge technologies, such as single-cell RNA sequencing (scRNA-seq), have propelled systems biology forward, revealing that many disease phenotypes are driven by networks of molecular interactions, thereby offering new avenues for drug development (Moss et al., 2022). For instance, in oncology, 53.3% of new drugs developed between 1946 and 1980 were either natural products or derivatives thereof, with a reduced but still significant proportion of 33.5% from 1981 to the present (Newman and Cragg, 2020). Similarly, the structural influence of NPs has been substantial in the field of anti-fibrotic treatments, leading to notable advancements (Henderson et al., 2020). These findings underscore the efficacy of NPs against fibrosis.

**FIGURE 1:**
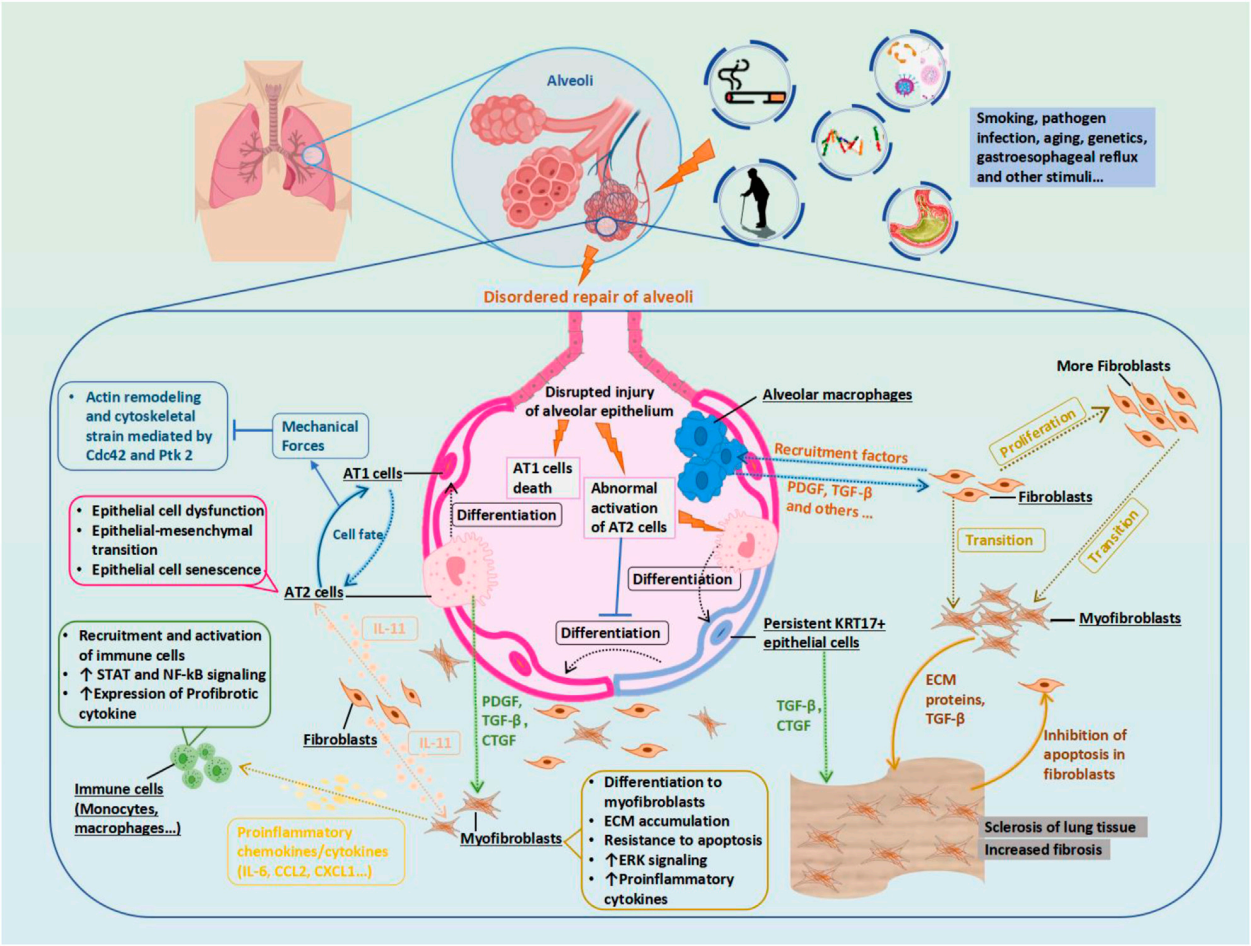
Potential Mechanism of Idiopathic Pulmonary Fibrosis (IPF) and Therapeutic Targets. Biophysical forces generated by normal respiration maintain AT1 cell identity and limit its differentiation into AT2 cells through the Cdc42 and Ptk 2 pathways. Epithelial injury signaling in the diseased alveoli triggers the secretion of IL-11 by fibroblasts, promoting fibroblast proliferation, migration, invasion, and myofibroblast differentiation. During the normal repair of the alveoli, AT2 cells differentiate into persistent KRT 17+epithelial cells as a transition state, from which they then differentiate into AT1 cells. Macrophages interact with other cells to remove debris without disrupting normal gas exchange in the alveoli. However, in the process of disordered repair of alveoli, AT1 cells die, AT2 cells are abnormally activated and produce PDGF and CTGF, and KRT17 + cells produce a large amount of connective tissue and activate TGF- β, promoting the proliferation of fibroblasts into myofibroblasts. Profibrotic alveolar macrophages are recruited, and their secreted PDGF promotes the activation and proliferation of fibroblasts and their differentiation into myofibroblasts. In the mutual positive feedback, the myofibroblasts secrete an excessive ECM, leading to a stiffening of the lung tissue and increased fibrosis. Abbreviations: AT1, type 1 alveolar cell; Cdc42, Cell division control protein 42 homolog; CTGF, connective tissue growth factor; ECM, extracellular matrix; KRT17, Keratin 17; IL11, interleukin 11; PDGF, Platelet-derived growth factor; Ptk2, Protein tyrosine kinase 2, also known as FAK1; TGF-β, Transforming growth factor beta.

**FIGURE 2:**
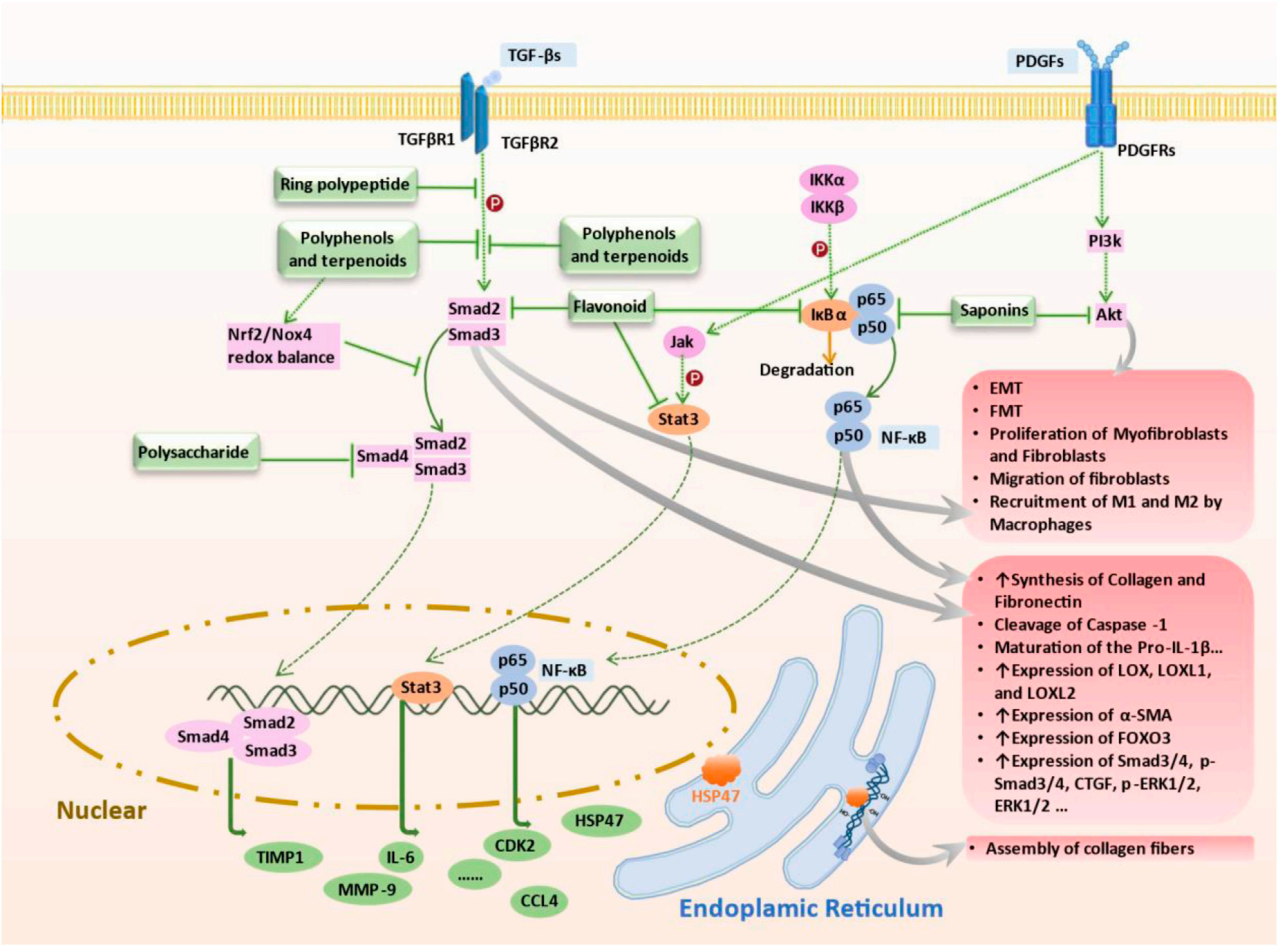
Mechanism of Targeted Therapy by Terrestrial-Derived Natural Products. Natural compounds from terrestrial sources combat pulmonary fibrosis via targeting key pathways: 1) Flavonoids inhibit TGF-β/Smad2/3 phosphorylation, attenuating fibrotic progression; 2) Cyclic peptides activate AMPK, reducing STING overexpression, 3) Polyphenols and terpenes inhibit TGF-β1 signaling, downregulate Smad3/4 and inhibit the promoting effect of HSP47 on collagen fiber formation to combat fibrosis; 4) Polysaccharides inhibit AUF1-mediated FOXO3 expression, reducing oxidative stress and fibrosis progression; 5) Flavonoids target NF-κB signaling in lung fibroblasts and IPF mouse models; 6) Saponins regulate NF-κB/p65 pathway in cell models; 7) Flavonoids inhibit TGF-β1 pathway via Jak2-Stat3/Stat1 signaling, hindering fibrosis; 8) Saponins suppress PI3K/AKT pathway and MMPs in bleomycin mouse models to alleviate fibrosis. These natural compounds demonstrate therapeutic potential in managing pulmonary fibrosis by modulating specific molecular pathways associated with the condition. Abbreviations: AKT, Protein kinase B (PKB), also known as Akt; AMPK, AMP-activated protein kinase; AUF1, AU-rich element RNA-binding factor 1; FOXO3, Forkhead box O3, also known as FOXO3 or FOXO3a; Jak2, Janus kinase 2; STAT3, Signal transducer and activator of transcription 3; PI3K, Phosphoinositide 3-kinases, also called phosphatidylinositol 3-kinases; TGF-β, Transforming growth factor beta; NF-κB, nuclear factor kappa B; HSP47, heat shock protein47.

**FIGURE 3:**
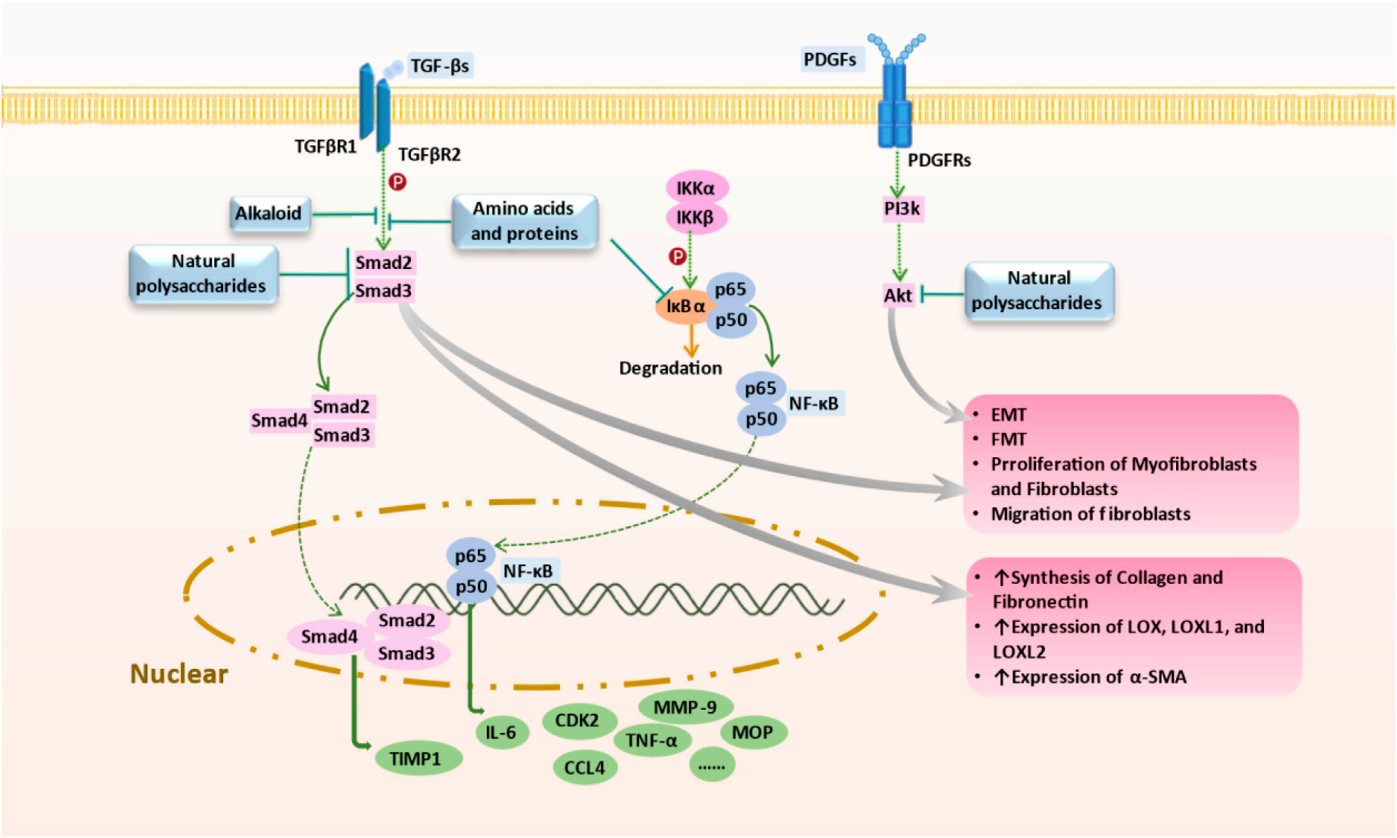
Mechanism of Targeted Therapy by Marine-Derived Natural Products. Marine-derived anti-pulmonary fibrosis compounds target key pathways: 1) Brown algal polysaccharides and deep-sea fungal alkaloids inhibit TGF-β/Smad signaling, reducing fibrotic progression; 2) Natural polysaccharides block NF-κB pathway, limiting EMT and collagen synthesis; 3) Polysaccharides inhibit PDGF-induced PI3K/AKT pathway, mitigating fibrosis progression; 4) Spirulina proteins decrease IL-6, TNFα, and MPO levels, potentially combating pulmonary fibrosis. Abbreviations: AKT, Protein kinase B (PKB), also known as Akt; EMT, Epithelial-to-mesenchymal transition; PDGF, MPO, Myeloperoxidase; Platelet-derived growth factor; PI3K, Phosphoinositide 3-kinases, also called phosphatidylinositol 3-kinases; TGF-β, Transforming growth factor beta; TNFα, Tumor necrosis factor alpha; NF-κB, nuclear factor kappa B.

PF results from dysregulated tissue repair following injury, particularly mediated by chronic inflammatory responses, which ultimately leads to fibrotic scarring. The effector cells implicated in PF are fibroblasts and myofibroblasts, which differentiate under the influence of local growth factors in IPF. Additionally, the excessive accumulation of extracellular matrix (ECM) components such as collagen disrupts mesenchymal integrity. ECM remodeling, involving the release of matrix metalloproteinases (MMPs), adversely affects alveolar ventilation. Thus, the activation and differentiation of local fibroblasts, the secretion of inflammatory mediators, and ECM synthesis are primary drivers of PF (Thannickal et al., 2004) (Figure 1). Various studies have validated the anti-inflammatory and anti-fibrotic potential of alkaloids (Liu et al., 2023), polysaccharides, flavonoids, peptides, terpenoids, and polyphenols (Ding et al., 2019). These compounds have demonstrated efficacy in inhibiting inflammatory responses, protecting against lipid peroxidation, suppressing mesenchymal cell activation and proliferation (Midgley et al., 2020), influencing the synthesis and secretion of pro-fibrotic factors (Li et al., 2017a), and regulating ECM synthesis and degradation, thereby offering therapeutic benefits for an anti-fibrotic treatment. Compounds such as baicalein, ginsenosides, natural polysaccharides (Chen et al., 2018; Chen et al., 2020), and flavonoids (Qian et al., 2018) have shown potential in inhibiting fibroblast proliferation and differentiation and reducing ECM synthesis by modulating various signaling pathways, including STAT3/miR-21/Spry1, Smad2/CTGF, Wnt/β-Catenin, and β-Catenin, thereby mitigating tissue fibrosis progression, as summarised in Figures 2, 3, and Tables 3–6.

**TABLE 3:** Summary of terrestrial natural products for the antifibrotic treatment of idiopathic pulmonary fibrosis.

Category	Origin (terrestrial)	Compound	Chemical Structure	Animal Disease model	Sample for pharmacological analyses	Target and signaling	References
Flavonoids	*Polygonum aviculare*	Juglanin	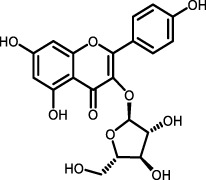	LPS-induced mouse model/bleomycin-induced mouse model	Ocular blood, pulmonary tissue cells, bronchoalveolar lavage fluid (BLAF)	Inhibited the NF-κB/STING signaling pathway to suppress TGF-β-induced collagen accumulation	(Sun et al., 2020)
	*Trifolium pratense L*	Biochanin-A	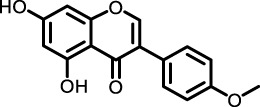	cell lines and primary cells from IPF patient//Bleomycin instillation in rat trachea	Human and rat lung tissue	Significantly attenuated the TGF-β1/BLM-mediated increase in TGF-β/Smad2/3 phosphorylation	Andugulapati et al. (2020)
	Mushroom *Inonotus Sanghuang*	Inonotus sanghuang extract	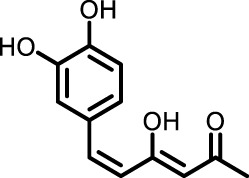	BLM-induce mouse IPF model	Lung tissue and BALF	Inhibited TGF-β-induced EMT-like phenotype and α-SMA expression, and decreased E-cadherin levels in A549 cells, reduce Smad2/3 phosphorylation and Snail expression	Su et al. (2021)
	*Salvia miltiorrhiza* (Danshen)	Tanshinone IIA	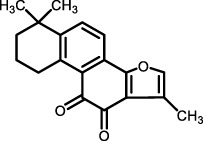	Silica-induced pulmonary fibrosis in Wistar rats	Lung tissue, and fibroblasts	Promoted the expression of nuclear Smad7, and inhibited the phosphorylation of Smad2 and Smad3 mediated by TβR1	Feng et al. (2020b)
	*Sorbus aucuparia*	Puerarin	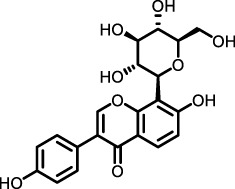	BLM-A5 IPF mouse model	Lower lobe of the left lung tissue	Inhibited TGF-β1 and IL6 through the JAK2-STAT3/STAT1 signaling pathway	Xue et al. (2021)
Saponins	*Arenaria kansuensis*	β-carbolines alkaloids	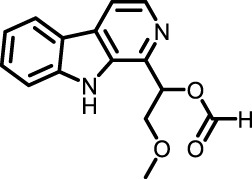	LPS-induced RAW264.7 cell model	Body weight, survival rate, lung tissue cells, bronchoalveolar lavage fluid	Inhibited phosphorylation of p65 to regulate the NF-κB/p65 pathway	Cui et al. (2019)
	Amaryllidaceae	Lycorine	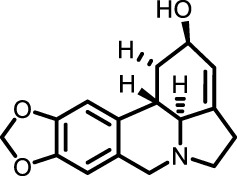	BLM-induced mouse model	Lung tissue, blood, bronchoalveolar lavage fluid	Inhibited cleavage of Caspase 1and pro-IL-1β/maturation of ASC-NLRP3 inflammasome complex formation	Liang et al. (2020)
	Ginseng	Total ginsenoside	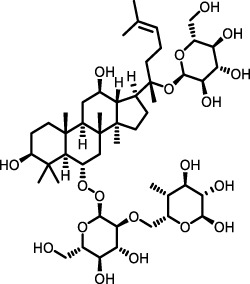	BLM-induced mouse model	Lung tissue	Inhibited the TGF-β1/Smad, MMP-2, -9 and TIMP1	Yang et al. (2019)
	*Astragalus membranaceus*	Astragaloside IV	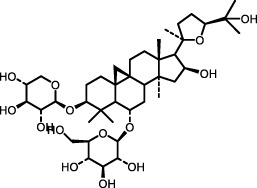	BLM-induced mouse model	Lung tissue	Inhibited the PI3K/Akt pathway	Qian et al. (2018) Li et al. (2017c)
Cyclic Peptides	Radix Pseudostellariae	Heterophyllin B	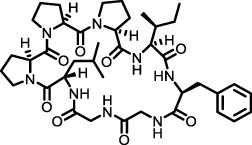	BLM-induced mouse model	Lung tissue, primary lung fibroblasts	Increased the activity of AMPK and thus lowered the STING expression	Shi et al. (2022)
Polyphenol-sand Terpenoids	Grapes, knotweed, and peanuts	Resveratrol	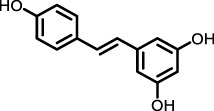	BLM-induced rat model	Lung tissue and BALF	Inhibited the expression of miR-21 and proteins of TGF-β1, α-SMA, Smad3/4, p-Smad3/4, CTGF, p-ERK1/2	(Wang et al., 2018) (Liu et al., 2023) (Ding et al., 2019)
	*Tripterygium wilfordii*	triptolide	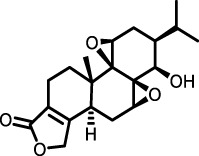	Mouse model of chest ion irradiation	Lung tissue and BALF	Inhibited recombinant protein TGF-β-induced overexpression of LOX, LOXL1, LOXL2, and thus the integrin-β1-FAK-YAP pathway	Lin et al. (2023)
	*Cymbopogon winterianus*	The essential oil of *Cymbopogon winterianus*	Eugenol (40.06%): 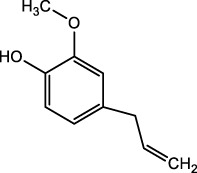 Geraniol (27.44%): 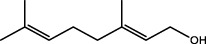 Citronellal (10.45%): 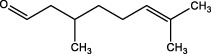	BLM-induced rat model	Lung tissue, blood, and BALF	Inhibited the expression of TGF-β1, and thus fibroblast-to-myofibroblast (FMT)	Tavares et al. (2021) (Ulanowska and Olas, 2021) (Mączka et al., 2020; Api et al., 2021)
	*Salvia miltiorrhiza*	Salvianolic acids	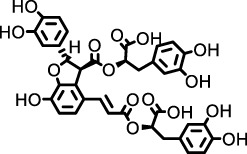	PQ-induced mouse model	Lung tissue	Interfering the Nrf2/Nox4 pathway and inhibited TGF-β1/Smad3 pathway	Liu et al. (2016), Du et al. (2016), Zhang et al. (2021)
	*Punica granatum*	Punicalagin	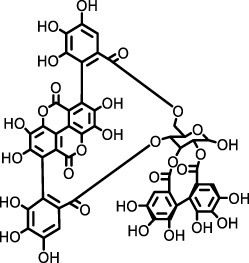	HSP47 expression system in *E. Coli*	*Escherichia coli*, collagen solution, drug screening library	Inhibited the promotion of collagen fiber formation by HSP47	Okuno et al. (2021)
	*Dryocosmus kuriphilus*	An ester of dehydrodigallic acid with two molecules of crenatin, also called chesnatin	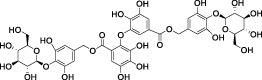	HSP47 expression system in *E. Coli*	*Escherichia coli*, collagen solution, drug screening library	Inhibited the promotion of collagen fiber formation by HSP47	Okuno et al. (2021)
	Green tea	Epigallocatechin-3-O-gallate	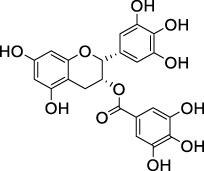	HSP47 expression system in *E. Coli*	*Escherichia coli*, collagen solution, drug screening library	Inhibited the promotion of collagen fiber formation by HSP47	Okuno et al. (2021)
Polysaccha-rides	*Cordyceps*	*Ophiocordyceps lanpingensis* polysaccharides (OLP)		BLM-induced mouse model	Lung tissue	Reduced the expression of myofibroblast marker α-SMA protein and the recruitment of macrophages M1 and M2	Zhou et al. (2020)
	*Dendrobium officinale*	Polysaccharides from *Dendrobium officinale*		BLM-induced mouse model	Lung tissue, blood and BALF	Inhibited the expression of Smad2/3 and pSmad2/3, and thus downregulated TGFβ1-Smad2/3 signaling	Chen et al. (2018)
	*Angelica sinensis*	Angelica polysaccharide		BLM-induced rat model and cell line from rat ATII cells (RLE-6TN)	Lung tissue	Inhibited AUF1-mediated FOXO3 protein expression levels, thereby alleviated oxidative stress	Qian et al. (2020)

While significant progress has been made in using small molecule NPs for liver and kidney fibrosis and lung cancer treatment, literature specifically addressing NPs directly targeting IPF remains sparse. This review aims to bridge this gap by examining the pathological effects of IPF and summarizing relevant NPs based on core pathological pathways and targets of anti-fibrotic disease. Utilizing literature from the Web of Science and PubMed databases (2013-up to date), we identified keywords such as “natural products,” “anti-pulmonary fibrosis,” and “drug screening.” We systematically screened the collected literature according to the sources and mechanisms of action of NP drugs, discussing the anti-pulmonary fibrosis processes of over 20 compounds from terrestrial and marine sources. Additionally, this review outlines various methods for establishing *in vivo* and *in vitro* disease models, techniques for analyzing disease markers or signaling pathways, and insights from multi-omics analyses, providing essential theoretical and technical references for the development of new anti-pulmonary fibrosis (pro)-drugs.

## 2 Overview of idiopathic pulmonary fibrosis

### 2.1 Characterization of idiopathic pulmonary fibrosis

Histologically, IPF is characterized by UIP patterning, in which the lung interstitium—a band-like tissue supporting alveoli—undergoes ECM deposition and architectural disorganization (Bonella et al., 2023). The pathological process typically initiates at the lung bases and periphery, where aberrant ECM accumulation progressively replaces functional parenchyma. This results in interstitial thickening, fibrotic alveolar septal collapse, terminal airway dilatation, and traction bronchiectasis/bronchiolectasis. Over time, persistent remodeling culminates in honeycombing—a hallmark of end-stage fibrosis—with irreversible structural distortion. As fibrotic tissue encroaches on alveoli, gas exchange becomes severely compromised, driving progressive respiratory dysfunction, clinical deterioration, and poor outcomes despite therapeutic interventions (George et al., 2019).

### 2.2 Pathogenesis and therapeutic targets of idiopathic pulmonary fibrosis

#### 2.2.1 Alveolar epithelial cell injury and abnormal repair

The etiology and pathogenesis of PF, particularly IPF, are largely undetermined, but notable advancements and consensus have been achieved in the field. IPF is generally believed to result from aberrant wound healing responses following damage to alveolar epithelial cells (AECs). Normally, AECs exhibit sophisticated and efficient repair mechanisms, with wound healing progressing through four distinct stages: coagulation/clotting, inflammatory cell migration, fibroblasts migration/proliferation/activation, and tissue remodeling and degradation (Raghu et al., 2022a; Spagnolo et al., 2021). However, factors such as cigarette smoking, subclinical infections, environmental pollutants, occupational exposures, chronic microaspiration of gastric content, abnormal lung microbiota composition, and genetic predisposition may cause repeated AEC injury (Bonella et al., 2023). This repeated injury leads to aberrant repair processes, excessively activating various inflammatory responses, repair pathways, and signaling pathways, resulting in the secretion of numerous cytokines.

Complex bidirectional interactions between epithelial cells, mesenchymal cells, and ECM subsequently induce a series of cellular events, including fibroblasts differentiation into myofibroblasts, migration, proliferation, and activation of AECs and fibroblasts, inhibition of apoptosis, and persistent abnormal ECM secretion. The continuous ECM deposition in the lung interstitium between alveoli and capillaries leads to irreversible structural changes and lung damage (Raghu et al., 2022a; Spagnolo et al., 2021). Within the lung epithelial tissue, alveolar epithelial type I (AT1) cells are the primary mediators of gas exchange, occupying most of the epithelial surface. Alveolar type II epithelial (AT2) cells act as progenitor cells for AT1, capable of differentiating into AT1 cells to renew and repair alveolar epithelial tissue while secreting surfactant, aiding in metabolic and immune functions. The differentiation of AT2 into AT1 is critical for maintaining normal lung function (Liu et al., 2022a; Moss et al., 2022; Herrera et al., 2018).

Alveolar epithelial wound repair is a complex, coordinated process. Persistent imbalance or injury at any stage of tissue repair can lead to fibrosis, progressing to various lung diseases. AT2 cells senescence, apoptosis, abnormal differentiation, and reduced abundance can impair alveolar epithelial repair and significantly contribute to PF. In IPF, AT2 cells senescence, apoptosis, abnormal differentiation, and decreased abundance are prevalent in the gas exchange regions of the lungs. Factors such as endoplasmic reticulum stress, mitochondrial dysfunction, and telomere shortening within these cells impede their ability to repair damaged epithelium effectively (Moss et al., 2022; Yao et al., 2021). Additionally, repetitive alveolar epithelial micro-injury, preventing AT2 cells from differentiating into AT1, results in abnormal alveolar epithelial regeneration and repair, increasing mechanical tension, which activates TGF-β signaling in AT2 cells (Moss et al., 2022). The aberrant TGF-β signaling induces the epithelial-mesenchymal transition (EMT), so that they acquire the characteristics of migration and secretion of ECM, directly promoting fibrosis (Wu et al., 2020) (Figure 1).

#### 2.2.2 Fibroblast-to-myofibroblast transformation and ECM deposition

The excessive transformation of fibroblasts into myofibroblasts during IPF progression is another critical factor. Under normal conditions, fibroblasts maintain ECM homeostasis. However, during acute injury, chronic inflammation, or repeated micro-injury, fibroblasts lose this balance and transform into myofibroblasts for repair. Myofibroblasts, characterized by enhanced proliferation and migration capabilities, secrete numerous cytokines and ECM, expressing proteins like alpha-smooth muscle actin (α-SMA), contributing to damage repair (Hinz and Lagares, 2020; Younesi et al., 2024). In IPF, excessive myofibroblast proliferation, apoptosis evasion, and invasiveness drive disease progression. Epithelial cell-secreted TGF-β promotes myofibroblast differentiation and proliferation, leading to further ECM deposition and accelerating disease progression. Inhibiting myofibroblast ECM secretion and contraction, as well as fibroblast-to-myofibroblast transformation (FMT), is a pivotal therapeutic approach for organ fibrosis (Hinz and Lagares, 2020; Mayr et al., 2024).

#### 2.2.3 MMP networks

ECM deposition is a hallmark of IPF, reflecting an imbalance between increased ECM synthesis and dysregulated degradation that culminates in irreversible scarring. At the molecular level, ECM degradation is mainly mediated by MMPs, the zinc-dependent endopeptidases secreted by macrophages, fibroblasts, NK cells, and neutrophils following tissue injury (Greenlee et al., 2007). Fibrotic scar tissues, primarily composed of cross-linked type I collagen fibers, is initially degraded by collagenases (e.g., MMP-1, MMP-2, MMP-8, MMP-13, MMP-14, MMP-15, and MMP-16), followed by gelatinases (e.g., MMP-2 and MMP-9) and other proteases (Sang, 1998; Karsdal et al., 2016). Beyond ECM degradation, MMPs also play critical roles in regulating inflammation and cellular behaviour by modulating growth factors, cytokines, chemokines, and cell surface receptors, thereby orchestrating a complex network of signaling events that ultimately drive IPF progression (Karsdal et al., 2016).

In IPF, dysregulated MMP expression and function drive disease progression through diverse mechanisms. For instance, MMP-3 promotes EMT by activating Wnt/β-catenin and TGF-β signaling pathways, while simultaneously inducing fibroblast proliferation and collagen deposition, as demonstrated by reduced fibrosis in MMP-3-knockout mice following bleomycin challenge (Pardo et al., 2016; Roque et al., 2020). Similarly, MMP-7, which is highly expressed in IPF lung tissue and plasma, is secreted by damaged AECs and drives fibrosis through multiple mechanisms, including cleaving basement membrane components such as E-cadherin and integrin β4, activating bioactive molecules including osteopontin and TGF-β, and promoting fibroblast activation and collagen deposition (Richards et al., 2012). Consequently, MMP-7 deficiency attenuates fibrosis, and its serum levels have emerged as a potential diagnostic biomarker for IPF (Richards et al., 2012; White et al., 2016).

Beyond these direct profibrotic actions, MMPs also modulate the fibrotic microenvironment through complex feedback loops with other cellular components. For example, MMP-9, produced by both AECs and Thy-1-negative fibroblasts, not only activates TGF-β to sustain a profibrotic cycle but also enhances fibroblast migration and collagen deposition (Kobayashi et al., 2014), thereby contributing to abnormal alveolar bronchiolization. Meanwhile, the circulating fibrocytes—CD45^+^/collagen^+^ bone marrow-derived cells—exploit the proteolytic activity of MMP-2, MMP-8, and MMP-9 to degrade ECM barriers, facilitating their migration into lung tissue and subsequent differentiation into myofibroblasts (Kobayashi et al., 2014; Espindola et al., 2021). Furthermore, MMPs critically modulate inflammation and immune responses that fuel fibrosis progression. Elevated MMP-8 levels in IPF bronchoalveolar lavage fluid (BALF) exacerbate neutrophil-mediated inflammation and fibrosis by cleaving anti-inflammatory factors like IL-10. Similarly, MMP-28 (Epilysin) regulates macrophage polarization, with knockout mice showing reduced M2 macrophage infiltration and attenuated fibrosis (Roque et al., 2020). These findings collectively underscore the multifaceted roles of MMPs in IPF pathogenesis, functioning not merely as ECM modifiers but as dynamic regulators of cellular crosstalk, immune responses, and signaling pathways that collectively drive fibrotic progression.

However, paradoxically, certain MMPs demonstrate context-dependent antifibrotic potential, suggesting protective roles under specific conditions. For instance, although MMP-1 efficiently degrades collagen, it is predominantly expressed in AECs during IPF, where it promotes epithelial repair and inhibits apoptosis—effects suppressed by the dominant profibrotic mediator TGF-β (Sang, 1998; Richards et al., 2012; Kobayashi et al., 2014; Link et al., 2025). Similarly, MMP-19 exerts protective effects by inhibiting fibroblast activation through prostaglandin E_2_ (PGE_2_)-mediated pathways and preserving non-fibrotic lung regions (Yu et al., 2012). These findings underscore the multifaceted roles of MMPs in IPF, warranting careful consideration in the development of targeted therapies.

Collectively, MMPs drive IPF by activating TGF-β, altering ECM biomechanics, and sustaining myofibroblast survival, making them therapeutic targets. However, their functional heterogeneity and organ-specific roles demand highly specific strategies. Challenges include safety concerns (e.g., impaired wound healing from long-term MMP inhibition) and functional redundancy, where MMP-2 or MMP-12 might compensate for MMP-9 inhibition, necessitating multi-target inhibitors or combination therapies (Greenlee et al., 2007; Sang, 1998; Karsdal et al., 2016; Pardo et al., 2016; Roque et al., 2020; White et al., 2016; Kobayashi et al., 2014; Espindola et al., 2021; Sun et al., 2020; Lin et al., 2023; Khan et al., 2024). Future research should integrate multi-omics to dissect dynamic MMP networks and advance precision-targeted or combinatorial approaches to improve IPF treatment.

#### 2.2.4 Collagen assembly and fibrogenesis: the key role of HSP47

HSP47 (Serpin H1) is indispensable for the efficient secretion, processing, fibril assembly, and deposition of collagen in the ECM. As an endoplasmic reticulum (ER)-resident, collagen-specific molecular chaperone, HSP47 binds to the Yaa-Gly-Xaa-Arg-Gly motif within procollagen triple helices via hydrophobic and hydrophilic interactions. This binding prevents misfolding and aggregation of procollagen and facilitates its transport to the Golgi apparatus (Okuno et al., 2021; Bellaye et al., 2021).

Clinical and preclinical evidence supports a critical role for HSP47 in fibrogenesis. Lung tissues from IPF patients show significantly elevated HSP47 expression, which positively correlates with collagen deposition (Okuno et al., 2021; Khan and Däinghaus, 2024). In bleomycin-induced murine models of pulmonary fibrosis, both pharmacological inhibition and siRNA-mediated silencing of HSP47 reduce collagen accumulation and improve lung architecture and function (Bellaye et al., 2021; Sakamoto et al., 2023). Moreover, elevated serum HSP47 levels in various interstitial lung diseases, including acute interstitial pneumonia, further underscore its potential as a biomarker for fibrotic progression (Sakamoto et al., 2023).

Furthermore, HSP47’s fibrogenic role is not isolated but intricately linked to key profibrotic signaling cascades, particularly those mediated by TGF-β. TGF-β1 upregulates HSP47 expression through the MAPK signaling pathway, thereby promoting excessive collagen synthesis (Khan and Däinghaus, 2024; Ito and Nagata, 2017). HSP47 stabilizes collagen to increase ECM stiffness, which further amplifies TGF-β signaling. This establishes a self-perpetuating cycle that exacerbates fibroblast activation and fibrotic progression. Additionally, the inflammatory cytokine IL-1β synergizes with TGF-β to activate heat shock factor 1 (HSF1), which transcriptionally upregulates HSP47, forming a pro-fibrotic positive feedback loop (Sakamoto et al., 2023). These findings collectively indicate that targeting HSP47—either directly or via its upstream regulatory pathways—may effectively disrupt pathological collagen metabolism, offering novel therapeutic opportunities for IPF management.

### 2.3 Molecular mechanisms and current research on drug therapy for idiopathic pulmonary fibrosis

The global prevalence of IPF has been rising, although some studies suggest a decline in overall mortality, potentially linked to reduced smoking rates, decreased use of immunosuppressants, and new therapies (Jeganathan et al., 2021). Clinically, pirfenidone and nintedanib, approved by the U.S. Food and Drug Administration (FDA) for IPF treatment, effectively slow the decline in forced vital capacity (FVC) and reduce mortality risk, though their mechanisms of action and safety profiles differ (Raghu et al., 2022a).

Pirfenidone is an orally administered synthetic compound with multiple mechanisms of action. At the cellular level, pirfenidone inhibits fibroblast activation and proliferation, including downregulation of genes related to fibroblast activation such as α-SMA (Maher, 2024; Nathan et al., 2017; Noble et al., 2011). In terms of anti-inflammatory effects, it suppresses the production of pro-inflammatory cytokines—including TNF-α, IL-1β, and IL-6—which are crucial contributors to the chronic inflammatory milieu in IPF (Conte et al., 2014; Jin et al., 2019). By reducing inflammatory cytokine release and alleviating oxidative stress–induced damage to lung epithelial cells, pirfenidone can mitigate the fibrotic process (Bonella et al., 2023; Solomon et al., 2023). In addition, it blocks the pro-proliferative effects of platelet-derived growth factor (PDGF) and fibroblast growth factor (FGF), thereby curtailing abnormal fibroblast activation and delaying disease progression (Conte et al., 2014; Jin et al., 2019). Despite these benefits, pirfenidone is associated with adverse events such as gastrointestinal intolerance and skin reactions, which are typically mild to moderate and often reversible upon dose adjustment (King et al., 2014; Behr et al., 2021; Spagnolo et al., 2021).

Nintedanib is a potent receptor tyrosine kinase inhibitor that targets multiple signaling pathways implicated in IPF. By blocking PDGF-BB/PDGFRβ signaling, it reduces fibroblast proliferation and migration (Maher, 2024; Lamb, 2021; Wollin et al., 2015). Additionally, it inhibits FGF/FGFR signaling, thereby diminishing fibroblast activation and ECM synthesis, and suppresses vascular endothelial growth factor (VEGF)/VEGFR signaling to lower vascular permeability and abnormal angiogenesis—indirectly limiting the fibrotic microenvironment (Lamb, 2021; Flaherty et al., 2019; Huang et al., 2017). Nintedanib also interferes with PI3K-Akt and other downstream pathways, inducing fibroblast apoptosis and restricting their survival. Furthermore, it exerts anti-inflammatory effects by inhibiting inflammatory mediators (e.g., IL-1β, IL-6) and reducing macrophage and neutrophil infiltration (Liu et al., 2022a; Huang et al., 2017). Collectively, by disrupting fibroblast proliferation, migration, and differentiation into myofibroblasts, nintedanib blocks fibrosis progression (Liu et al., 2022a; Spagnolo et al., 2021). Nevertheless, its side effects—including gastrointestinal issues and liver function abnormalities—necessitate careful monitoring during treatment (Flaherty et al., 2019; Richeldi et al., 2014).

In advanced stages of IPF, comorbidities such as pulmonary hypertension and acute exacerbations significantly worsen symptoms (Maher, 2024; George et al., 2019; Richards et al., 2012). The annual incidence of acute exacerbations is about 10%, often leading to high mortality—up to 3 months post-exacerbation (Spagnolo et al., 2021; Richards et al., 2012; Solomon et al., 2023; Teague et al., 2022). Given the lack of curative treatments, substantial resources have been invested in research and clinical trials to develop new therapies. However, numerous trials, especially in Phase II and III, have failed to achieve their primary endpoints (Liu et al., 2022a; Bonella et al., 2023; Solomon et al., 2023). For example, BG00011 is a humanized anti-α_v_β_6_ IgG1 monoclonal antibody designed to inhibit α_v_β_6_ binding to latent TGF-β, thereby blocking TGF-β activation following tissue injury or inflammation. After positive results in Phase IIa, a Phase IIb study evaluated the efficacy and safety of a 56 mg (approximately 0.7 mg/kg) flat dose of BG00011 compared with placebo in a larger IPF cohort, both with and without background therapy (pirfenidone or nintedanib). However, long-term BG00011 exposure induced acute exacerbations and inflammation, leading to reduced survival rates, and the drug was ultimately discontinued from clinical development (Raghu et al., 2022b). Alongside these trial failures, other major challenges in developing novel IPF therapies include inadequate animal models, insufficient interventions targeting epithelial repair, and a limited understanding of how cellular senescence contributes to fibrosis (Mora et al., 2017). These unresolved issues underscore the complexity of IPF and highlight the urgent need for novel, mechanism-based therapeutic strategies.

Given these setbacks in novel drug development, alternative strategies such as drug repurposing have gained traction in IPF research. For example, metformin, an oral antidiabetic agent, has demonstrated potential in reversing fibrosis in a bleomycin-induced mouse model. The study showed that metabolically active and apoptosis-resistant fibrotic regions in IPF patients and mouse models exhibited low expression of AMP-activated protein kinase (AMPK). Metformin, an AMPK activator, inhibits TGF-β1-induced myofibroblast differentiation by promoting AMPK expression, leading to myofibroblast deactivation and apoptosis, reduction in the expression of type I collagen, fibronectin (FN), and α-SMA in fibroblasts, and downregulation of ECM protein levels, thereby slowing or reversing PF (Rangarajan et al., 2018). Similarly, Saracatinib, a selective Src family kinase inhibitor, has shown considerable promise for IPF treatment (Li et al., 2020). Computational drug repositioning revealed an inverse transcriptomic correlation between Saracatinib’s signature and IPF disease signatures, suggesting its antifibrotic potential. Mechanistically, Saracatinib inhibits TGF-β-induced Smad3 phosphorylation, thereby suppressing key fibrotic markers—such as α-SMA, type I collagen, and plasminogen activator inhibitor-1 (PAI-1)—in human lung fibroblasts, while reversing EMT-related pathways (Ahangari et al., 2022). In both bleomycin- and Ad-TGF-β-induced murine pulmonary fibrosis models, saracatinib significantly reduced lung hydroxyproline (an indicator of collagen deposition), improved lung compliance, and attenuated radiographic abnormalities, with efficacy comparable or superior to nintedanib and pirfenidone (Hu et al., 2014; Baselga et al., 2010). *Ex vivo* experiments using precision-cut lung slices from IPF patients and mouse models further confirmed saracatinib’s ability to downregulate fibrotic genes, reduce collagen accumulation, and decrease α-SMA-positive areas. Transcriptomic analyses indicated that saracatinib reversed dysregulated IPF pathways—including EMT, immune responses, and ECM organization—all of which relied on Src kinase activity (Ahangari et al., 2022). Collectively, these findings underscore the promise of repurposing existing drugs for IPF treatment, potentially accelerating the translation of effective therapies into clinical practice.

While drug repurposing offers convenience and safety advantages, providing a new avenue for IPF treatment, clinical testing remains essential. Although pirfenidone and nintedanib can decelerate disease progression, they do not stabilize or improve lung function, halt or reverse disease progression, or cure the disease. Given their side effects, tolerance, and safety issues, developing new drugs remains challenging. Therefore, advancing clinical trials and exploring new screening methods for potential drugs and molecular targets are crucial to addressing the current limitations in IPF treatment and diagnostics.

## 3 Therapeutic potential of natural products for idiopathic pulmonary fibrosis

NPs, derived from both terrestrial and marine sources, have significantly influenced the field of IPF treatment. This chapter categorizes NPs into terrestrial and marine sources, examining their distinct chemical structures and biological activities. Terrestrial NPs encompass a variety of classes including polyphenols, flavonoids, isoflavonoids, saponins, terpenoids, biphenyls, alkaloids, and cyclic peptides (Tables 3, 4). Similarly, marine environments have yielded numerous pharmacologically active NPs (Tables 5, 6). These compounds demonstrate a wide spectrum of chemical structures and biological activities, targeting multiple mechanisms and holding considerable potential for drug development. In contemporary medical research, the structural diversity and biological activities of NPs have garnered substantial attention in developing anti-IPF drugs. The following sections detail the mechanisms by which various NPs act on different targets, illustrating their prospective applications in antifibrotic drug research.

**TABLE 4:** Summary of terrestrial natural products regarding the efficacy and medication use.

Clinical drug name	Efficacy	Medication use
Biochanin-A	On widely used bleomycin induced pulmonary fibrosis in rats, inflammatory markers (ALP and LDH) and inflammatory cells (neutrophils count) were significantly reduced in BCA treatment compared to BLM control samples (Andugulapati et al., 2020)	SRB assay revealed, 50% cell death was observed at a concentration of 73.55 ± 0.3 µM, 79.73 ± 0.22 µM and 103.02 ± 0.3 µM in NHLF, DHLF and LL29 cells respectively (Andugulapati et al., 2020)
Tanshinone IIA	In a silicosis rat model, Tan IIA alleviates silicosis lung fibrosis and is effective in inhibiting EMT and TGF-β1/Smad signaling induced by silica in lung epithelial cells (Feng et al., 2020b)	Tan IIA caused proliferation inhibition at 40 μM in A549 and HBE cells (Feng et al., 2020b)
Juglanin	In bleomycin-treated mice, after 20 days of treatment, the survival rate of Jug administration-treated mice was 60%, which was much higher than that of the control group, which was approximately 10% (Sun et al., 2020)	Jug was treated to mice from days 1–21 via daily oral gavage at 80 mg/kg according to previous study (Sun et al., 2020)
β-carboline alkaloids	The survival rate of mice in BLM group is only 20% on day 15, however, treatment with β-carbolines-rich Part1 at the dose of 100 and 150 mg/kg could elevate the survival rate up to 50%. And the body weight of mice significantly increased after Part1 treatment (Cui et al., 2019)	For bleomycin-treated mice,100 and 150 mg/kg were effective, while 50 mg/kg was ineffective (Cui et al., 2019)
Lycorine	The results demonstrated that the LYC treatment ameliorated BLM-induced pulmonary fibrosis and inflammation in mic (Liang et al., 2020)	The result of MTT assay showed that lycorine itself, even at a concentration of 5 μM, did not affect the growth of RAW264.7 cells obviously after 24 h treatment (Kang et al., 2012)
Total ginsenosides	At 28 days after BLM injection, the mice had breathing abnormalities, decreased activity, and lack of luster in their hair. In the Model group, the pulmonary coefficient was significantly increased. Compared with the model control, the condition of the mice improved and the pulmonary coefficient was markedly reduced in the TG group (Yang et al., 2019)	80 mg/kg/day, 40 mg/kg/day, and 160 mg/kg/day TG were effective (Yang et al., 2019)
Salvianolic acids	Administration of bleomycin resulted in a significant increase in the lung index (777% ± 0.72%). The SalB (0.45 mg/kg) group exhibited a marked reduction in the lung index (4.21% ± 0.24%) after 28 days of treatment. It also reduced the levels of hydroxyproline (HYP), collagen type I (Col-1), tissue factor (TF), and related coagulation factors induced by BLM, while simultaneously decreasing the expression of plasminogen activator inhibitor-1 (PAI-1), thereby delaying the progression of pulmonary fibrosis in rats (Zhang et al., 2021)	The drug was administered via aerosolization. The delivery rate was 0.60 ± 0.06 mg/min while the total delivered dose was 4.57 ± 0.04 mg. The total exhalation dose was 2.40 ± 0.20 mg (Zhang et al., 2021)
Resveratrol	The resveratrol treatment groups, particularly those receiving medium and high doses, significantly improved pulmonary fibrosis scores, reduced the lung index and oxidative stress markers, and decreased collagen deposition. Furthermore, resveratrol inhibited TGF-β1-induced proliferation and differentiation of pulmonary fibroblasts and exerted its antifibrotic effects by modulating the TGF-β/Smad/ERK signaling pathway (Liu et al., 2023)	High dose: 100 mg/kg, medium dose: 50 mg/kg, and low dose: 25 mg/kg were administered continuously for 28 days. In the cell-based experiments, the 3T6 fibroblast cell line was treated with resveratrol at concentrations of 2.5 mg/mL, 5 mg/mL, and 10 mg/mL for 24 h (Liu et al., 2023)
Triptolide	Triptolide was shown to reduce the mRNA and protein expression levels of α-SMA, type I collagen, fibronectin, and vimentin, while also inhibiting the migration and transdifferentiation of pulmonary fibroblasts. Moreover, triptolide exhibited inhibitory effects on the TGF-β/SMAD signaling pathway both *in vivo* and *in vitro* (Lin et al., 2023)	Triptolide (16 μg kg^-1^ day^-1^) were intragastrically administered for 35 days since 7th day after BLM administration (Lin et al., 2023)
Essential oil from *Cymbopogon winterianus* (EOCW)	Oral administration of EOCW alleviated the progression of pulmonary fibrosis in a bleomycin-induced mouse model, with a reduction in myofibroblast differentiation that may be attributed to the inhibition of TGF-β expression (Tavares et al., 2021)	Oral administration of EOCW at 100 and 200 mg/kg daily for 28 days significantly ameliorated lung tissue injury (Tavares et al., 2021)
*Ophiocordyceps lanpingensis* polysaccharides (OLP)	In a mouse model of bleomycin (BLM)-induced pulmonary fibrosis, oral administration of OLP was found to inhibit fibrosis development by reducing macrophage recruitment, preserving alveolar function, and suppressing collagen deposition (Zhou et al., 2020)	Oral administration of OLP at doses of 0.3 g/kg, 0.5 g/kg, and 1.0 g/kg daily for 3 weeks (Zhou et al., 2020)
Heterophyllin B from Radix Pseudostellariae	Heterophyllin B potentially exerts protective effects against BLM-induced pulmonary fibrosis by inhibiting TGF-β/Smad2/3 signaling and AMPK-mediated STING expression (Shi et al., 2022)	From day 8 to day 21 of BLM injection, heterophyllin B were orally administered daily (20 mg/kg) (Shi et al., 2022)

**TABLE 5:** Summary of marine natural products for the antifibrotic treatment of idiopathic pulmonary fibrosis.

Category	Origin (marine)	Compound	Study model	Animal disease model	Sample for pharmacological analyses	References
Polysacch-arides	Phaeophyta	Low molecular weight fucoidan	BLM-treated mouse model and TGF-treated A549 cells	Lung tissue and BALF	Downregulated the TGF-β/Smad and PI3K/AKT signaling pathways, reduced oxidative stress and inflammation, and significantly reduced the expression of key factors such as COL2A1, β-catenin, TGF-β, TNF-α and IL-6 in lung tissue	(Wu et al., 2021) (Dong et al., 2022) (Yu et al., 2018)
Saponins and Alkaloids	Deep-sea fungus *Tricho-derma* sp. MCCC 3A01244	Trichocarboline A	Human lung fibroblast cell line HFL1	HFL1 cell	Potential anti-pulmonary fibrosis effect by inhibiting TGF-β/Smad signaling, reducing ECM deposition *in vitro*	Hao et al. (2022)
Natural Amino Acids and Proteins	*Spirulina platensis*	Phycocyanin	BLM-induced mouse model	Serum and lung tissue	Attenuated vimentin upregulation and E-cadherin downregulation, reduced interleukin 6 (IL-6), tumor necrosis factor-α (TNF-α) and myeloperoxidase (MPO) levels, and inhibited the TLR2-MyD88- NF-κB pathway	Li et al. (2017b)
	*Eucheuma*	EZY-1	BLM-induced mouse model	Lung tissue	Inhibited PI3K p85 subunit, ERK1, p38MAPK and c-Abl, and thus regulated PI3K-Akt-mTOR, Rac1-PAK2-cAb1 and MAPK pathway. And EZY-1 significantly inhibited the phosphorylation of Smad2 and Smad3 in mouse lung tissue, indicating that it interfered with TGF-β/Smad signaling pathway	Yu et al. (2019)

**TABLE 6:** Summary of marine natural products regarding the efficacy and medication use.

Marine natural product	Efficacy	Medication use
Low Molecular Weight Fucoidan	In 8-week-old C57BL/6 mice, the infiltration of neutrophils and macrophages in lung tissue induced by radiation was significantly reduced. The expression levels of inflammatory cytokines (such as TIMP-1, CXCL1, MCP-1, MIP-2 and IL-1Ra) in pleural effusion were reduced (Dong et al., 2022; Wu et al., 2021)	LMWF had no significant effect on the activity of A549 cells, and no concentration or time-dependent cytotoxicity was observed. No significant toxicity observed at high doses (up to 640 μg/mL *in vitro*). *In vivo* experiment, no toxicity was observed at a high dose (100 mg/kg) (Dong et al., 2022; Wu et al., 2021)
Trichocarboline A(from *Trichoderma* sp. MCCC 3A01244)	Suppresses TGF-β/Smad signaling pathway, reducing ECM deposition *in vitro* (Hao et al., 2022)	In HFL1 cells, collagen accumulation was significantly inhibited at a concentration of 10 μM, with an effect close to pirfenidone. Low cytotoxicity was detected at effective concentration (10 μM) (Hao et al., 2022)
Phycocyanin	Reduces pulmonary fibrosis by modulating TLR2-MyD88-NF-κB pathway and enhancing antioxidant defenses (Li et al., 2017b)	Non-toxic; safe in animal models. Good water solubility; bioavailability enhanced through peptide derivatives
EZY-1	Inhibits multiple signaling pathways (TGF-β/Smad, PI3K-Akt-mTOR, MAPK), reducing fibrosis in bleomycin-induced mouse models. EZY-1 inhibited the phosphorylation of ERK and p38MAPK, preventing the progression of idiopathic pulmonary fibrosis by inhibiting the MAPK signaling pathway activated by TGF-β	High safety profile; no acute toxicity observed at doses up to 5 g/kg (Yu et al., 2019)

### 3.1 Overview of targeted signaling pathways of natural products

The NPs exhibit therapeutic potential in IPF by modulating key signaling pathways involved in inflammation, myofibroblast activation, oxidative stress, and ECM deposition. Among these, the NF-κB, TGF-β/Smad, AMPK, Nrf2, and PI3K/Akt pathways stand out as key regulators. Below, we summarize how each pathway contributes to IPF pathogenesis and highlight representative NPs that target these molecular mechanisms.

#### 3.1.1 NF-κB signaling

The NF-κB pathway is a critical mediator of inflammation and is aberrantly activated in various diseases, including breast, lung, and colon cancers (Baker et al., 2011). When AECs are damaged by oxidative stress, infection, or mechanical injury, NF-κB can be triggered via pattern recognition receptors (e.g., TLR4) or pro-inflammatory factors (TNF-α, IL-1β). This activation enhances the expression of TNF-α, IL-6, CCL2, and MCP-1, leading to intensified local inflammation and exacerbation of fibrosis development (Dong et al., 2022). Additionally, immune cells (e.g., macrophages, neutrophils) activated by NF-κB secrete cytokines (e.g., TNF-α, IL-1β), which further stimulate NF-κB in AECs, amplifying the inflammatory cascade in IPF (Baker et al., 2011; Li et al., 2017b).

Identifying NF-κB inhibitors is therefore crucial for antifibrotic therapy, for which multiple NPs have shown promise. For example, phycocyanin (Li et al., 2017b) and β-carboline alkaloids found in Xuelingzhi (Cui et al., 2019) can both inhibit NF-κB, thereby suppressing the recruitment and activation of inflammatory cells. Additionally, juglanin, extracted from crude “*Polygonum aviculare*”, has also been shown to inhibit the NF-κB pathway (Dong and Yuan, 2018). Similarly, phycocyanin can inhibit the abnormal activation of AECs through the TLR2-MyD88-NF-κB signaling mediated by TLR2 (Li et al., 2017b), thus reducing the secretion of ECM components and pro-inflammatory cytokines/chemokines, such as IL-6, CCL2, and CXCL1 (Li et al., 2017a). Furthermore, β-carboline alkaloids from snow fungus exhibit anti-inflammatory activity by inhibiting NF-κB signaling (Cui et al., 2019). These natural compounds demonstrate considerable potential in modulating NF-κB signaling, thereby offering therapeutic benefits in attenuating inflammation-driven fibrotic progression in IPF.

#### 3.1.2 TGF-β1/smad pathway

The TGF-β signaling pathway is a principal therapeutic target in IPF, as it is prominently upregulated and activated during fibrotic progression. Specifically, TGF-β1/Smad signaling significantly contributes to IPF pathogenesis by facilitating the activation, proliferation, and differentiation of epithelial cells and collagen-producing myofibroblasts, ultimately exacerbating ECM deposition and fibrosis development (Wu et al., 2021; Peng et al., 2022). In murine fibrosis models, overexpressed TGF-β binds to type II TGF-β receptor (TβRII) forming a heterodimer with TβRI, leading to excessive activation and phosphorylation of Smad2/3 proteins. Activated Smad2/3 then translocate into the nucleus, initiating transcriptional regulation of numerous fibrotic genes, triggering fibroblast phenotype transformation, promoting EMT, and significantly enhancing ECM accumulation (Peng et al., 2022; Derynck and Zhang, 2003; Massagué, 2012). Furthermore, TGF-β also activates the PI3K-Akt and Erk pathways, facilitating EMT, collagen accumulation, and inflammatory responses, further exacerbating fibrosis development (Wu et al., 2021).

Considering the crucial pathological role of the TGF-β/Smad pathway, the therapeutic potential of various natural products in IPF treatment has been explored and reported. For example, compounds such as Biochanin-A (BCA) (Andugulapati et al., 2020), *Inonotus sanghuang* extract (ISE) from ethyl acetate fraction (Su et al., 2021), salvianolic acid B (Liu et al., 2016), total ginsenosides (TG) (Yang et al., 2019), *Dendrobium officinale* (Chen et al., 2018), the amino acid EZY-1 (Yu et al., 2019), and low molecular weight fucoidan (LMWF) (Wu et al., 2021) were shown to effectively inhibit this signaling in bleomycin-induced mouse models of pulmonary fibrosis. Additionally, *in vitro* studies confirm that LMWF (Wu et al., 2021) alleviates EMT and fibrotic phenotype by simultaneously blocking TGF-β/Smad and PI3K/Akt pathways in A549 cells, indicating multifaceted therapeutic effects. Furthermore, tanshinone IIA (Tan IIA) (Feng et al., 2020a), derived from traditional Chinese medicine *Salvia miltiorrhiza* (Danshen), robustly suppresses TGF-β1/Smad signaling, inhibiting collagen I, collagen III, and α-SMA expression, effectively reducing fibrosis severity in a silica-induced pulmonary fibrosis model using Wistar rats. In another example, triptolide (Lin et al., 2023) exhibited antifibrotic effects in a mouse model of chest ion irradiation-induced lung injury. Moreover, trichocarboline A, a β-carboline alkaloid from the deep-sea fungus *Trichoderma* MCCC 3A01244 (Hao et al., 2022), was further tested to found to reduce pulmonary fibrosis by downregulating Smad2 and Smad3 phosphorylation, thereby directly inhibiting TGF-β/Smad signal transduction in human lung fibroblast cell lines (HFL1). These findings collectively underscore the cross-species and multi-model efficacy of natural products in targeting the TGF-β/Smad pathway, highlighting their promise as therapeutic agents for IPF.

#### 3.1.3 AMPK pathway

AMPK is an energy sensor activated under energy-deficient conditions to regulate cellular metabolism (Hardie et al., 2012). Upon activation, it inhibits energy-consuming pathways and promotes energy-generating processes, thereby maintaining cellular homeostasis. Additionally, AMPK also promotes autophagy, facilitating the clearance of damaged organelles and proteins—an essential process for delaying or preventing fibrosis (El-Horany et al., 2023). In the bleomycin-induced mouse model of pulmonary fibrosis, the AMPK activator metformin reverses established fibrosis by suppressing TGF-β1-driven collagen and FN production, enhancing mitochondrial biogenesis, restoring myofibroblast susceptibility to apoptosis, and promoting autophagy-mediated ECM degradation (Rangarajan et al., 2018; Kheirollahi et al., 2019). Moreover, heterophyllin B, isolated from Radix Pseudostellariae, ameliorates fibrosis by activating AMPK, which in turn suppresses the TGF-β1/Smad2/3 signaling and downregulates STING expression. These actions collectively reduce collagen deposition and restoring energy metabolism (Shi et al., 2022).

#### 3.1.4 Nrf2 pathway

During IPF pathogenesis, persistent oxidative damage contributes to epithelial injury, fibroblast activation, and ECM deposition (El-Horany et al., 2023; Hybertson et al., 2011), while the Nrf2 signaling pathway plays a crucial role in mitigating oxidative stress. *In vivo* and *in vitro* studies demonstrate that activation of Nrf2 reduces fibrotic progression by upregulating antioxidant enzymes such as heme oxygenase-1 (HO-1) and NAD(P)H quinone dehydrogenase 1 (NQO1), thereby countering reactive oxygen species (ROS) accumulation in lung tissues (Dong et al., 2022; Zhang et al., 2018). Notably, Nrf2 activation inversely regulates NADPH oxidase 4 (NOX4), a key ROS-generating enzyme implicated in TGF-β-induced myofibroblast differentiation and epithelial cell apoptosis (Liu et al., 2016). This antagonistic relationship highlights Nrf2’s critical role in regulating oxidative stress and restraining the fibrotic cascade in IPF (Hybertson et al., 2011).

Building on these findings, several NPs have been shown to exert antifibrotic effects by activating the Nrf2 pathway *in vitro* and *in vivo*. For instance, Salvianolic acid B was shown to attenuate paraquat-induced pulmonary fibrosis by restoring the Nrf2/NOX4 redox balance, where Nrf2 activation suppressed NOX4 expression and downstream TGF-β1/Smad3 signaling (Liu et al., 2016). And tanshinone IIA (Tan IIA) (Feng et al., 2020a), from *S. miltiorrhiza*, enhances Nrf2-mediated expression of antioxidant enzymes, thereby reducing fibrosis severity in silica-induced pulmonary fibrosis models. Similarly, geraniol (Tavares et al., 2021), a component of essential oil from *Cymbopogon winterianus*, activates Nrf2, leading to downregulation of pro-oxidant mediators and attenuation of fibrotic progression. Additionally, LMWF (Dong et al., 2022) alleviated bleomycin-induced fibrosis through Nrf2-mediated suppression of NOX4 and oxidative stress markers. These findings collectively highlight the therapeutic potential of targeting the Nrf2/NOX4 axis to disrupt oxidative stress-driven fibrotic cascades in IPF, and natural products targeting this pathway offer promising therapeutic potential for mitigating oxidative stress and halting fibrosis progression.

#### 3.1.5 PI3K/Akt pathway

The PI3K/Akt signaling pathway is a central regulator of cell growth, proliferation, and survival. Importantly, elevated PI3K/Akt activity has been observed in IPF lung tissues and in bleomycin-induced pulmonary fibrosis models, underscoring its pathophysiological relevance (Wang et al., 2022a). In the fibrotic lung, activation of PI3K/Akt promotes EMT, enhances fibroblast survival, and stimulates ECM production, thereby exacerbating fibrosis progression (Qian et al., 2018).

Recently, several NPs have been identified to exert antifibrotic effects by suppressing PI3K/Akt signaling. Compounds like Astragaloside IV (Qian et al., 2018), a natural saponin from *Astragalus membranaceus*, and LMWF (Wu et al., 2021) have been identified as effective inhibitors of the PI3K/Akt signaling cascade in bleomycin-treated mice, thereby exerting a beneficial inhibitory effect on ECM deposition and fibrosis progression. These findings underscore the therapeutic promise of targeting the PI3K/Akt pathway with natural compounds to attenuate fibrosis in IPF.

Altogether, these interconnected pathways—NF-κB, TGF-β1/Smad, AMPK, Nrf2, and PI3K/Akt—underscore the multifactorial nature of IPF pathogenesis. NPs, through their potential to simultaneously modulate multiple pathways, emerge as promising candidates for developing innovative antifibrotic therapies. In the subsequent section, we will systematically examine the anti-pulmonary fibrotic mechanisms of over 20 bioactive compounds derived from both terrestrial (Section 3.1) and marine (Section 3.3) sources and one nature product analogue (Section 3.4). This comprehensive analysis will encompass: (1) experimental models employed in their pharmacological evaluation, including both *in vivo* animal systems and *in vitro* cell-based platforms; (2) key pathological markers analyzed in these studies, such as collagen deposition indicators, inflammatory cytokines, and EMT biomarkers; and (3) molecular targets validated through contemporary pharmacological approaches.

### 3.2 Terrestrial natural products and their mechanisms

#### 3.2.1 Flavonoids

Biochanin-A (BCA), an organic isoflavone isolated from the leaves and stems of *Trifolium pratense* L and many other herbs of Chinese medicine (Yan et al., 2021), has been demonstrated to mitigate PF in various cell lines, including LL29, normal human lung fibroblasts (NHLF), and diseased human lung fibroblasts (DHLF). BCA achieves this by modulating the TGF-β/Smad3 pathway, thereby ameliorating the fibrosis cascade response and inhibiting the expression of fibrosis markers such as FN-I during the EMT. *In vitro* scratch assays revealed that BCA dose-dependently attenuates TGF-β-induced cell migration. In a bleomycin-induced pulmonary fibrosis model in Wistar rats, a 14-day oral administration of BCA significantly reduced lung inflammation, infiltration of inflammatory cells, expression of inflammatory markers, collagen deposition, and fibrosis markers in lung tissues. BCA treatment also markedly decreased the expression of phospho-smad3, α-SMA, and FN-I, along with histopathological abnormalities in lung tissue (Andugulapati et al., 2020). These findings underscore BCA’s potential application in modulating pathways related to PF and managing disease progression.

Tanshinone IIA (Tan IIA), a lipophilic component derived from *S. miltiorrhiza* (Danshen), significantly inhibits the EMT process in silica-induced pulmonary fibrosis models by suppressing the TGF-β1/Smad signaling pathway (Feng et al., 2020a). In Wistar rats exposed to silica, intraperitoneal injection of Tan IIA effectively reduced collagen I, collagen III, and α-SMA expression, diminishing the extent of PF. *In vitro* studies demonstrated that Tan IIA treatment increased nuclear expression of Smad7, inhibited Smad2 and Smad3 phosphorylation by TβR1, and reduced the nuclear accumulation of phosphorylated Smad3, thus impeding ECM biosynthesis and myofibroblast differentiation. Moreover, Tan IIA mitigated oxidative stress induced by silica. Activation of the Nrf2 signaling pathway in A549 and human bronchial epithelial (HBE) cell lines further enhanced Tan IIA’s antifibrotic effects by modulating EMT and TGF-β1/Smad pathway activation (Feng et al., 2020a). Additionally, puerarin (Pue), a compound derived from *Sorbus aucuparia*, exhibits synergistic effects with Tan IIA in combating fibrosis progression (Xue et al., 2021).

In an IPF mouse model administered bleomycin-A5 intratracheally, Tan IIA and Pue combined therapy significantly reduced the expression of fibrosis markers and macrophage infiltration, improving lung function and survival rates. *In vitro* experiments in NIH-3T3 cells revealed that the combination treatment downregulated α-SMA expression and inhibited JAK2, STAT3, and STAT1 signaling pathways, reducing fibroblast activation and migration induced by TGF-β1 and IL6, thereby suppressing fibrosis progression through the JAK2-STAT3/STAT1 pathway. Juglanin (Jug, kaempferol-3-O-α-L-arabinofuranoside), a compound isolated from crude “*Polygonum aviculare*”, inhibits the STING pathway, effectively counteracting TGF-β-induced collagen accumulation. Elevated Sting expression was observed in bleomycin-induced mouse lung tissues, suggesting that bleomycin may induce pulmonary fibrosis through STING activation. Juglanin treatment significantly reduced Sting expression, ameliorating TGF-β-induced collagen accumulation and highlighting its potential application in treating fibrotic diseases (Williams et al., 2020).

#### 3.2.2 Saponins and alkaloids

β-carboline alkaloids, extracted from Snow Fungus, exhibit anti-inflammatory effects by inhibiting the secretion of cytokines MCP-1, IL-6, TNF-α, and IL-1β in LPS-induced RAW264.7 cells, thereby mitigating inflammation (Cui et al., 2019). These alkaloids regulate the NF-κB/p65 pathway by inhibiting p65 phosphorylation, thereby curbing inflammation initiation. In TGF-β1-induced A549 cells, β-carboline alkaloids prevented the suppression of E-cadherin by TGF-β1, reduced mesenchymal and fibrotic markers such as α-SMA and Vimentin, and slowed the EMT process, thereby impeding PF progression.

Lycorine (LYC), an alkaloid from *Amaryllidaceae* plants, significantly reduced alveolar collapse and collagen accumulation in bleomycin-induced pulmonary fibrosis in mice, lowering fibrosis marker levels including hydroxyproline (HYP). Flow cytometry revealed that LYC could reverse macrophage reduction *in vivo*. Real-time PCR demonstrated that LYC blocked bleomycin-induced expression of type I collagen, Acta2, and Fn, and reduced α-SMA and FN levels, improving bleomycin-induced pulmonary fibrosis. LYC also exerted anti-inflammatory effects by inhibiting Caspase-1 cleavage and pro-IL-1β maturation, alleviating fibrosis and inflammation. Molecular docking and SPR analysis showed that LYC inhibited NLRP3 inflammasome activation and pyroptosis by binding to the PYD domain of ASC, suppressing ASC-NLRP3 interaction, thus exhibiting anti-inflammatory effects and improving fibrosis (Liang et al., 2020).

Total ginsenosides (TG), chemical components from ginseng, have demonstrated therapeutic efficacy against IPF. TG treatment improved bleomycin-induced pulmonary fibrosis in mice, reducing interstitial fibrosis, collagen deposition, and alveolar wall destruction. TG downregulated TGF-β1, α-SMA, Smad2, and Smad3 expression while upregulating Smad7, inhibiting fibroblast proliferation and abnormal ECM deposition via the TGF-β1/Smad pathway. Additionally, TG’s protective effect against IPF may involve the MMP system, as TG significantly downregulated MMP-2, MMP-9, and tissue inhibitor of metalloproteinase-1 induced by bleomycin, potentially disrupting basement membrane integrity and mitigating fibroblast invasion (Yang et al., 2019).

#### 3.2.3 Polyphenols

Polyphenolic compounds, a diverse class with extensive biological activities, include phenolic acids, flavonoids, stilbenes, lignans, and tannins. These compounds have shown significant efficacy in alleviating fibrotic disease symptoms (Du et al., 2016). Salvianolic acids, notably Salvianolic acid A (Sal A) and Salvianolic acid B (Sal B) from *S. miltiorrhiza*, exhibit robust antioxidant activity. Nrf2 is critical for cellular protection against oxidative stress, and NOX4, closely linked to the TGF-β1/Smad3 pathway in lung fibroblasts, is essential for regulating the myofibroblast phenotype in IPF. Sal B modulates the Nrf2/Nox4 redox balance and TGF-β1/Smad3 signaling, alleviating oxidative stress in paraquat-poisoned mice and showing potential therapeutic effects on PF (Zhang et al., 2021). Sal B also reduces hydroxyproline (HYP), type I collagen, tissue factor (TF), and related coagulation factors, exerting antifibrotic and anticoagulant effects (Liu et al., 2016). Given Sal’s therapeutic potential, efforts are being made to develop a new inhalable dry powder formulation to enhance bioavailability, which is currently less than 5% orally (Jiang et al., 2021).

Resveratrol (Res), a polyphenolic compound, mitigates oxidative damage and fibrosis by leveraging its antioxidant properties, thereby preventing PF *in vivo*. MicroRNA-21 (miR-21) amplifies TGF-β1 signaling, exacerbating PF progression. In bleomycin-induced rat models, Res inhibited miR-21 by downregulating c-Jun and c-Fos, blocking the MAPKs/AP-1 pathway (Wang et al., 2018). Res also modulated fibrosis-related proteins FN, COL1 A1, COL3 A1, α-SMA, TIMP-1, and MMP-2, 9, and 13, inhibiting PF progression through the TGF-β/Smad/ERK pathway. Additionally, Res alleviated PM2.5-induced lung inflammation and fibrosis by inhibiting autophagy and NLRP3 inflammasome activity, reducing IL-1β in BEAS-2B cells (Ding et al., 2019). These properties suggest that resveratrol holds promise for PF treatment.

#### 3.2.4 Terpenoids

Triptolide, a bioactive diterpene from *Tripterygium wilfordii*, was shown to downregulate mRNA and protein expression of α-SMA, type I collagen, FN, and vimentin, inhibiting lung fibroblast migration and transformation. Triptolide reduced TGF-β1-induced overexpression of LOX, LOXL1, and LOXL2, as well as total LOX activity, diminishing oxidative stress and altering endopeptidase expression such as MMP2, MMP9, MMP13, and MMP14. Immunofluorescence showed Triptolide inhibited the Integrin-β1-FAK-YAP pathway, reducing nuclear YAP1 content and inhibiting fibrotic gene transcription (Lin et al., 2023). These findings support Triptolide’s potential as an antifibrotic agent. Essential oil from *Cymbopogon winterianus* (EOCW), containing eugenol, geraniol, and citronellal, has anti-inflammatory and antioxidant properties. Eugenol enhances Nrf2 expression, geraniol inhibits 5-lipoxygenase and nitric oxide, and citronellal reduces COX-2 and prostaglandin-E2 (PGE2) expression. EOCW alleviated pulmonary fibrosis progression in bleomycin-induced mice, likely due to TGF-β expression inhibition (Tavares et al., 2021). These properties make EOCW a candidate for PF treatment, warranting further investigation into its dosage and mechanisms.

#### 3.2.5 Natural polysaccharides

*Ophiocordyceps lanpingensis* polysaccharides (OLP), extracted from the edible fungus *Ophiocordyceps lanpingensis*, show therapeutic potential against PF. In bleomycin-treated mice, OLP reduced pro-inflammatory protein MCP-1, decreased macrophage recruitment, and downregulated related cytokines and myofibroblast markers like α-SMA. OLP preserved alveolar function and inhibited collagen production, mitigating bleomycin-induced pulmonary fibrosis (Zhou et al., 2020). *Dendrobium officinale*, an *Orchidaceae* herb, produces polysaccharides (PDO) with mild anti-inflammatory and strong antifibrotic effects. Oral PDO reduced E-cadherin and α-SMA expression and decreased type I collagen and FN synthesis in bleomycin-treated rats. PDO downregulated Smad2/3 and pSmad2/3 expression, alleviating myofibroblast formation and proliferation by inhibiting TGFβ1-Smad2/3 signaling (Chen et al., 2018). Angelica sinensis polysaccharide (ASP) from *Angelica sinensis* roots inhibits lncRNA DANCR in bleomycin-treated rat lung tissue and type II alveolar epithelial cells (RLE-6TN), reducing oxidative stress and PF severity. ASP reversed collagen deposition and α-SMA upregulation induced by TGF-β1, and restored E-cadherin expression (Qian et al., 2020). Polysaccharides’ complex composition and mechanisms necessitate further research to validate their pharmacological activity and support drug development.

#### 3.2.6 Cyclic peptides

Heterophyllin B from Radix Pseudostellariae exhibits anti-inflammatory activity by promoting AMPK activation and inhibiting STING, thereby reducing TGF-β1-induced abnormal proliferation of MLE-12 cells and overexpression of STING in bleomycin-treated mice. Heterophyllin B inhibited TGF-β1 and Smad2/3 phosphorylation, and reduced myofibroblast markers α-SMA and COL-1 in lung tissues, demonstrating anti-pulmonary fibrosis effects. It offers significant therapeutic potential, as shown in bleomycin-induced pulmonary fibrosis models treated with oral heterophyllin B (Shi et al., 2022).

### 3.3 Marine natural products and their mechanisms

#### 3.3.1 Natural polysaccharides

LMWF, a distinctive sulphated polysaccharide derived from seaweed through radical degradation, has a molecular weight of approximately 8,100 Da, with fucose as its primary monosaccharide. Wu et al. investigated the antifibrotic effects and mechanisms of LMWF using both *in vivo* and *in vitro* models (Wu et al., 2021). Protein blot analyses in lung cancer A549 cells (CCL-185, ATCC, Rockville, MD, US) were conducted via the MTT method. Lung tissues from different experimental groups of mice were collected for pathological evaluation through HE and Masson’s trichrome staining, histological, and immunohistochemical analysis. Serum IL-6 levels were measured using ELISA kits, and bronchoalveolar lavage fluid (BALF) was analyzed for lavage fluid and protein concentrations using BCA kits. Results demonstrated that LMWF treatment in bleomycin-induced pulmonary fibrosis mice decreased TGF-β, TNF-α, and IL-6 expression in lung tissue, mirroring the effects of nintedanib in reducing inflammatory cytokine accumulation. Furthermore, LMWF attenuated phosphorylation activation of the PI3K/AKT signaling pathway induced by TGF-β1 in A549 cells, lowering β-catenin and COL2A1 expression, and preventing EMT. In the TGF-β/Smad pathway, LMWF downregulated TGF-β1, Smad2/3, and Smad4 expression, thereby inhibiting pathway activity and curbing TGF-β1-induced EMT progression. These findings suggest that LMWF has significant therapeutic potential in inhibiting human IPF by mitigating inflammation and EMT processes (Dong et al., 2022; Wu et al., 2021).

Dong et al. also evaluated LMWF extracted from Laminaria japonica via radical degradation, focusing on its effects on bleomycin-treated male C57BL/6J mice and TGF-treated A549 cells, with an additional assessment of its antioxidant properties (Dong et al., 2022). Pathological changes and collagen deposition in lung tissues of bleomycin-induced mice were evident from H&E, Masson’s trichrome, and Sirius red staining. GSH, MDA, and SOD levels in lung tissues were measured using kits, and hydrogen peroxide content was assessed with enzyme biosensors. LMWF treatment reduced MDA and 8-iso-PGF2α levels while increasing SOD, GSH, HO-1, and NQO1 concentrations, thereby preventing oxidative stress damage and attenuating pulmonary fibrosis by enhancing antioxidant enzyme protein expression. *In vitro*, LMWF regulated antioxidant factors in lung fibrosis mouse lung tissue, reducing oxidative stress and fibrosis. Immunofluorescence, protein blotting, and immunohistochemistry showed that LMWF at 50 mg/kg and 100 mg/kg doses upregulated Nrf-2, HO-1, and NQO1 expression in a dose-dependent manner, inhibiting fibrosis. High doses (100 mg/kg) showed no toxicity in A549 cells, aligning with *in vivo* results, indicating LMWF’s high safety profile (Dong et al., 2022).

A separate study investigated the effects of fucoidan obtained via enzymatic hydrolysis on the infiltration of inflammatory cells in lung tissues of irradiated 8-week-old male C57BL/6 mice. Fucoidan significantly reduced neutrophil and macrophage infiltration in irradiated lung tissues. Cytokine array analysis and EIA confirmed that fucoidan lowered the expression levels of inflammatory cytokines such as TIMP-1, CXCL1, MCP-1, MIP-2, and IL-1Ra in pleural effusion of irradiated mice. *In vivo*, these cytokines were linked to radiation-induced lung fibrosis, selectively activating macrophages and neutrophils. Thus, fucoidan administration reduced cytokine expression in pleural effusion and decreased collagen expression in fibroblasts, correlating with reduced inflammatory cell infiltration in lung tissues. This finding suggests potential fucoidan-based therapies for preventing lung fibrosis in clinical settings (Yu et al., 2018). Overall, fucoidan, due to its wide availability and high safety profile, shows promise as a therapeutic agent for treating PF, with no observed toxic side effects at high doses (100 mg/kg) (Dong et al., 2022; Wu et al., 2021).

#### 3.3.2 Alkaloids

From the deep-sea fungus *Trichoderma* MCCC 3A01244, 25 compounds were isolated, among which Trichocarboline A, a β-carboline alkaloid, exhibited significant biological activity. At a 10 μM concentration in HFL1 cell lines, Trichocarboline A demonstrated low cytotoxicity and robustly inhibited TGF-β1-induced collagen accumulation. CCK8 assays showed a notable reduction in cellular collagen deposition. Mechanistic studies indicated that Trichocarboline A downregulated phosphorylation levels of Smad2 and Smad3, suppressing the TGF-β/Smad signaling pathway. This inhibition curtailed TGF-β1-induced expression of FN, proliferating cell nuclear antigen (PCNA), and α-SMA in HFL1 cells, reducing ECM deposition. Trichocarboline A binds to Smad4, hindering TGF-β/Smad pathway signaling, and downregulates transcription of fibrosis-related genes. As a newly identified β-carboline alkaloid, Trichocarboline A emerges as a lead compound for the development of more effective antifibrotic drugs (Hao et al., 2022).

#### 3.3.3 Natural amino acids and proteins

EZY-1, a 16-amino acid peptide derived from the edible seaweed *Eucheuma*, showed significant inhibitory effects on bleomycin-induced pulmonary fibrosis in mice. Using protein chip analysis and *in vitro* pull-down combined with LC-MS/MS, potential EZY-1 target proteins were screened. *In vitro* analysis identified ERK, Akt, raptor, SHP2, PDGFR, β-catenin, and vitronectin as potential targets. EZY-1 notably inhibited Smad2 and Smad3 phosphorylation in lung tissues of bleomycin-induced mice, suggesting interference with the TGF-β/Smad signaling pathway. Additionally, EZY-1 inhibited the phosphorylation of ERK and p38MAPK, blocking IPF progression through inhibition of the TGF-β-activated MAPK signaling pathway. EZY-1 also significantly reduced collagen fiber deposition and proline hydroxylation levels, lowered malondialdehyde (MDA) levels, and increased superoxide dismutase (SOD) and glutathione peroxidase (GSH-Px) activity, offering protection against oxidative damage. It inhibited tyrosine phosphorylation of proteins such as PI3K p85, ERK1, p38MAPK, and c-Abl, and demonstrated anti-fibrotic and anti-inflammatory properties superior to pirfenidone. EZY-1 exhibited high safety with no acute toxicity effects at a 5 g/kg dosage, indicating its promise as a safe and effective drug for IPF treatment (Yu et al., 2019).

Phycocyanin (PC), a light-harvesting protein from *Spirulina platensis*, was studied by Li et al. in C57 BL/6 wild-type (WT) mice and toll-like receptor (TLR) 2-deficient mice treated with PC for 28 days post-bleomycin exposure. PC significantly reduced PF markers, including hydroxyproline (HYP), vimentin, surfactant-associated protein C (SP-C), fibroblast-specific protein-1 (S100A4), and α-SMA, while increasing E-cadherin and podoplanin expression. PC also reduced early-stage inflammation-related proteins IL-6, TNF-α, and MPO. It improved the pathological state of PF by protecting type I alveolar epithelial cells and inhibiting EMT. In bleomycin-treated mice, PC reduced TLR2 pathway gene expression, with limited effects in TLR2-deficient mice, suggesting that PC mediates antifibrotic effects via the TLR2-MyD88-NF-κB pathway. Future exploration of this pathway may establish PC as a novel antifibrotic agent (Li et al., 2017b).

### 3.4 Natural product-like compound YX-2102 and its mechanisms

YX-2102, a pyrano [2,3-b]pyridine derivative identified through virtual screening from a synthetic in-house library of “natural product-like” compounds, acts as a highly selective cannabinoid receptor 2 (CB2R) agonist (Liu et al., 2022b). Functionally, it alleviates PF by suppressing M1 macrophage polarization and inflammation, while upregulating CB2R expression in AECs to inhibit TGF-β1-induced EMT. Mechanistically, YX-2102 modulates the Nrf2-Smad7 pathway, thereby blocking TGF-β/Smad signaling. *In vivo* studies demonstrate its efficacy in reducing bleomycin-induced lung injury and fibrosis with manageable toxicity. Its synthetic accessibility and structural tunability highlight its potential as a novel therapeutic agent for IPF (Liu et al., 2022a).

## 4 Outlook

### 4.1 Challenges and limitations

Despite their structural diversity and multi-target potential, NPs face significant barriers in clinical translation for IPF. Intrinsic challenges include low abundance of active compounds (e.g., resveratrol at <0.1% in grape skins) (Liu et al., 2023; Ding et al., 2019), necessitating high doses (e.g., 200 mg/kg for LMWF) (Dong et al., 2022; Wu et al., 2021) that strain natural sourcing and purification efforts. Structural complexity—such as macrolide stereoisomerism—hampers synthesis scalability, while poor pharmacokinetics (e.g., curcumin’s <1% oral bioavailability) (Wang et al., 2022b) limit therapeutic utility. Mechanistic ambiguity compounds these issues: most NPs lack fully defined targets (Flaxman et al., 2024; Wang et al., 2022a), and their polypharmacology, though potentially advantageous for multifactorial diseases like IPF, risks off-target effects (Meng et al., 2018). For instance, baicalin’s dual TGF-β/Wnt inhibition was only linked to EP300 histone acetylation in 2023, decades after its initial discovery (Williams et al., 2020; Tao et al., 2018). These challenges are exacerbated by IPF’s complex pathogenesis, complicating target prioritization (Spagnolo et al., 2021). While NPs like salvianolic acid show preclinical antifibrotic activity, their undefined targets and pharmacokinetics hinder clinical adoption—a stark contrast to approved synthetics like pirfenidone (2,403 mg/day) (Noble et al., 2011) and nintedanib (300 mg/day) (Richeldi et al., 2014), which themselves face tolerability limitations (Bonella et al., 2023; Raghu et al., 2022a).

### 4.2 Technology-driven solutions

Emerging interdisciplinary strategies are reshaping NP drug discovery. High-throughput phenotypic screening platforms now integrate CRISPR-edited organoids with ImageXpress systems (Fraietta and Gasparri, 2016), enabling single-cell resolution analysis of α-SMA suppression and reducing lead compound identification to 2 weeks (Nelson et al., 2024; Palano et al., 2020). In the construction of disease models, gene engineering techniques can be used to precisely modify animal genomes, creating animal models that more closely resemble the pathological processes of human pulmonary fibrosis. For instance, using CRISPR-Cas9 technology to knock-in or knock-out genes related to pulmonary fibrosis in mice can cause symptoms similar to human pulmonary fibrosis (Vazquez-Armendariz and Tata, 2023). These animal models can more realistically reflect the effects of drugs *in vivo*, providing reliable experimental evidence for preclinical drug research.

In target optimization, high-throughput screening can be applied to establish a screening model for inhibitors based on the core pathway of pulmonary fibrosis (TGF-β). Fluorescent reporter gene systems targeting TGF-β receptors (ALK5), Smad proteins, or downstream effector molecules (such as CTGF) can be designed. For instance, HEK293 cell lines can be constructed, and Smad binding element (SBE)-luciferase reporter plasmids can be transfected to screen for compounds that inhibit TGF-β-induced fluorescence signals. A study in 2022 used this model to screen out a novel ALK5 inhibitor, TP-008, which reduced collagen deposition in mouse pulmonary fibrosis models by 70% (Hanke et al., 2020). Alternatively, dual-reporter cell lines for E-cadherin (epithelial marker) and vimentin (interstitial marker) can be constructed to quantify EMT severity through changes in fluorescence ratios (Luttens et al., 2025). Additionally, multi-parameter screening based on cell phenotypes can be performed using AI image analysis systems to assist high-content imaging (HCI), simultaneously quantifying α-SMA expression, cell migration (Transwell assays), and collagen contraction (3D collagen gel models), automatically identifying myofibroblast morphology changes (such as pseudopod retraction), significantly enhancing the efficiency of screening in drug development (Weisbart et al., 2024; Seal et al., 2025). It can be seen that high-throughput screening is expected to evolve from single-target screening to multidimensional systems pharmacology in the research and development of anti-pulmonary fibrosis drugs.

Concurrently, advanced target deconvolution techniques like thermal proteome profiling (TPP) allow global analysis of thermal stability shifts across 7,000+ proteins (Savitski et al., 2014), as demonstrated by the discovery that CDC42 inhibitors stabilize β-catenin/E-cadherin complexes to suppress renal fibrosis—a mechanism with cross-organ relevance for IPF (Hu et al., 2024). For such multi-omics data, including proteomics and metabolomics, artificial intelligence technology can integrate genomics and transcriptomics (He et al., 2023). By deeply mining and analyzing these data, potential targets closely related to the development of pulmonary fibrosis can be identified, such as genes, proteins, and metabolites (Mann et al., 2021). For example, analyzing large-scale gene expression data from patients with pulmonary fibrosis and healthy individuals to identify differentially expressed genes and determine possible drug targets.

Furthermore, artificial intelligence–based computational approaches further accelerates progress: This strategy proved instrumental in identifying saracatinib as a multi-pathway inhibitor via TGF-β/WNT network analysis (Ahangari et al., 2022), and AlphaFold2-predicted NP-target interactions guided the redesign of salvianolic acid into derivative C25, which exhibits 12-fold improved solubility and 8-fold greater inhibition of type Ⅰ collagen (Noor et al., 2023; Zeng et al., 2022; Yang et al., 2023). These technologies may collectively bridge the gap between NP complexity and clinical applicability.

### 4.3 Clinical translation prospects and emerging frontiers

NPs demonstrate unique advantages in IPF therapeutic development, particularly in early-stage intervention and precision medicine (Wang et al., 2022a). Andrographolide elicits its anti-pulmonary fibrotic effect in preclinical models by halting the progression of EMT via affecting fibroblasts (Karkale et al., 2018), while preclinical studies have shown that curcumin combined with pyrifene can reduce the dose of pyrifene by 50% and enhance the inhibitory effect of α_v_β_6_ integrin (Raghu et al., 2022b; Wang et al., 2022b). Epigenetic modulation strategies are gaining traction, as evidenced by a selective HDAC6 inhibitor that durably suppresses pro-fibrotic genes like FN1 and IL-11, maintaining efficacy for 28 days post-treatment (Lin et al., 2023; Ng et al., 2019). Looking ahead, synthetic biology platforms are poised to address scalability challenges (Zhao et al., 2023); artemisinic acid production in engineered yeast now costs $350/kg (Paddon et al., 2013), a model applicable to high-value antifibrotic NPs like cordycepin (Zhou et al., 2020). Integration of single-cell spatial omics with lung-on-chip systems will further map cell-type-specific responses, enabling personalized regimens (Sisodia et al., 2023; Chen et al., 2023; Huang et al., 2024), underscoring their enduring potential in antifibrotic discovery.

## 5 Conclusion

This review has summarized NPs from marine and terrestrial sources, highlighting their promising antifibrotic activities through various mechanisms. Categories such as polyphenols, terpenes, alkaloids, and polysaccharides have been discussed, with polysaccharides showing slow progress due to complex and diverse mechanisms. These NPs exert preventive and therapeutic effects on PF and IPF by modulating pathways like the TGF-β1-mediated Smad pathway, Nox4-Nrf2 pathway, NF-κB pathway, and AMPK pathway. Additionally, the integration of NPs with emerging epigenetic modulators and precision medicine approaches offers novel strategies to overcome current treatment limitations. These findings underscore the vital role of natural products in developing next-generation anti-fibrotic therapies, providing new sources and reliable references.
